# Survey of U.S. broiler establishments on *Campylobacter* Performance standards for parts

**DOI:** 10.1016/j.psj.2025.105924

**Published:** 2025-09-30

**Authors:** Rafael E. Rivera, Harshavardhan Thippareddi, Sanjay Kumar, Manpreet Singh

**Affiliations:** a Department of Poultry Science, University of Georgia, Athens, GA 30602, USA; b Quality Technical Services and Food Safety, Niagara Bottling, Diamond Bar, CA 91765, USA; c Department of Food Science & Technology, University of Georgia, Athens, GA 30602, USA

**Keywords:** *Campylobacter*, Survey, Performance standard, Prevalence

## Abstract

*Campylobacter* is a major foodborne pathogen linked to poultry consumption and causes gastrointestinal illness. The U.S. Department of Agriculture, Food Safety and Inspection Service (USDA-FSIS) regulates pathogen control through performance standards targeting prevalence reduction at the processing operations. After revising *Campylobacter* performance standards in 2019, USDA-FSIS discontinued public reporting of testing results and categories. A survey of broiler processors evaluated *Campylobacter* post-harvest interventions and control strategies for chicken parts which was subsequently adopted by the U.S. broiler industry. A majority (62 %) of processing establishments were monitored for *Campylobacter* and 74 % have an established control program for *Campylobacter* for chicken parts. In 2021, 49 % of establishments met the USDA-FSIS *Campylobacter* performance standards for chicken parts, while 68 % met internal establishment sampling. Based on USDA-FSIS sampling, 38 % reported prevalence under 10 % in chicken parts, whereas 60 % of establishments reported prevalence under 10 % resulting from internal establishment sampling. Prevalence under 10 %, likely meets the USDA-FSIS threshold of 7.7 % for *Campylobacter* performance for parts. Notably, plant processing capacity did not influence prevalence. Over 90 % of establishments used peroxyacetic acid as an antimicrobial intervention for chicken parts. In response to the introduction of *Campylobacter* performance standards for chicken parts, the poultry processing industry has channeled investments into targeted interventions to mitigate contamination risks.

## Introduction

*Campylobacter* is a leading cause of zoonotic bacterial gastrointestinal infection in the United States. Symptoms of campylobacteriosis include diarrhea, fever, cramps, nausea, and vomiting (CDC, 2022a). The illness is typically self-limiting, with antimicrobial treatment reserved for severe cases (Wagenaar et al., 2013). Complications such as irritable bowel syndrome, transient paralysis, and arthritis may occur, and campylobacteriosis is also lined to Guillain-Barre syndrome (Williams et al., 2021).

According to the Foodborne Diseases Active Surveillance Network (FoodNet), approximately 20 annual cases of campylobacteriosis are diagnosed per 100,000 individuals, though most cases go undiagnosed or unreported. The Center for Disease Control and Prevention (CDC) estimates 1.5 million *Campylobacter* infections occurs yearly in the U.S. (CDC, 2022a). Poultry products are a primary source of *Campylobacter*. Over 80 % of non-dairy foodborne illnesses were attributed to chicken, other seafood (such as shellfish) and turkey (Berghaus et al., 2013; Ellis-Iversen et al., 2012; IFSAC, 2021; Overesch et al., 2020). *Campylobacter* infections from poultry products alone cost $6.9 billion annually in medical expenses and reduced quality of life (Scharff, 2020).

Many species of poultry, especially commercial broilers and turkeys, carry high levels of *Campylobacter* (*C. jejuni* and *C. coli*) in their gastrointestinal tract as part of their normal microbial flora (Sahin et al., 2015). The *Campylobacter* prevalence ranges from 23.6 to 60.6 % in flocks, with concentration of 3.63-5.89 log MPN/g (Berghaus et al., 2013). Other studies report higher prevalence (45-100 %) and with concentrations (3-7 log CFU/g) due to environmental factors like contaminated poultry litter, feed, drinking water, rodents, insects, and poor worker hygiene (Alter et al., 2005; Berrang et al., 2007; Potturi-Venkata et al., 2007; Stern et al., 2001) (Golden and Mishra, 2020; Sahin et al., 2015; Wagenaar et al., 2013). Transportation further spreads *Campylobacter* to the feathers and skin (Sahin et al., 2015; Seliwiorstow et al., 2016; Wagenaar et al., 2013). correlating with contamination levels at the processing plant (Berghaus et al., 2013). While effectiveness of pre-harvest interventions are unpredictable and inconsistent, post-harvest antimicrobial interventions at various processing steps remain critical to remove *Campylobacter* on chicken carcasses (DeVillena et al., 2022; Hwang and Singer, 2020; Williams et al., 2021).

The U.S. Department of Agriculture Food Safety and Inspection Service (USDA-FSIS) develops and monitors food safety regulatory standards for poultry. The USDA-FSIS (1996) Pathogen Reduction: Hazard Analysis and Critical Control Points rule (PR: HACCP Rule) established pathogen reduction, leading to decline in *Campylobacter* prevalence from 88.2 % (1995) to 18.3 % (2019) on chicken carcasses (Williams et al., 2021). However, infection rates have plateaued post- HACCP (CDC, 2022b).

In 2010, the USDA-FSIS announced the *Campylobacter* performance standard for chicken and turkey carcasses, followed by performance standards for parts and comminuted poultry in 2016 (USDA-FSIS, 2010, 2015a; Williams et al., 2021). Microbiological verification testing results were used to categorize poultry processing establishments based on *Campylobacter* prevalence levels. Subsequent performance standards for poultry parts (2016) faced challenges, as revised sampling/ enrichment method (e.g., neutralizing buffered peptone water; nBPW) for rinsing of the whole carcasses/ chicken parts resulted in a altered detection rates (USDA-FSIS, 2019). The USDA-FSIS revised the *Campylobacter* performance standards and discontinued reporting on testing results and categories until the microbiological verification testing were revalidated (USDA-FSIS, 2019).

The regulatory approach has mainly focused on reducing and/or eliminating foodborne pathogens at the processing establishment level. Currently, *Campylobacter* reduction is heavily addressed through antimicrobial chemical interventions. Poultry processors adjust antimicrobial interventions to meet performance standards (Wideman et al., 2016). Post-chill dip tanks and sprayers have been evaluated for their efficacy as antimicrobial interventions in chicken parts. Immersion of whole chicken or parts was shown to be more effective at reducing *Campylobacter* populations compared to spray application resulting from longer contact time and improved surface coverage (Gonzalez et al., 2021; Laranja et al., 2023). Chlorine has long been used as a processing aid in poultry processing, but its effectiveness is very limited. Chlorine’s disinfection capacity is highly dependent on chill water pH and the amount of organic matter in chillers (Chen et al., 2020). Chlorine, typically used as sodium hypochlorite, has been replaced by peroxyacetic acid (PAA; (DeVillena et al., 2022; Kataria et al., 2020). PAA provides a strong oxidizing function that disrupts the permeability of cell membranes and alters protein synthesis (Oyarzabal, 2005). PAA has emerged as a popular choice due to its overall effectiveness in reducing *Campylobacter*. When used at high concentrations, PAA can provide over 2.0 log CFU/mL reduction in *Campylobacter* counts (Vaddu et al., 2021; Wideman et al., 2016). Chen et al. (2014) reported that PAA solutions as low as 700 ppm can lower *Campylobacter* by 1.5 log CFU/g with residual effect during storage without affecting organoleptic qualities. Other approved antimicrobials that are used to a lesser degree are cetylpyridinium chloride (CPC), trisodium phosphate (TSP), acidified sodium chlorite (ASC), and chlorine dioxide (ClO_2_) (Chen et al., 2014; Oyarzabal, 2005; Zhang et al., 2018).

The USDA-FSIS conducted a risk assessment to estimate the effects of the proposed raw chicken parts and comminuted performance standards on public health (USDA-FSIS, 2015b) and reported that 46 % of U.S. chicken processing plants will not meet the new performance standards for parts. Further, a 30 % reduction in *Campylobacter* illnesses can be achieved if 40 % of chicken processing establishments failing the *Campylobacter* performance standards for Not-Ready-To-Eat Comminuted Poultry become compliant and a 32 % reduction in *Campylobacter* illnesses can be achieved if 50 % of chicken processing establishments failing the *Campylobacter* parts performance standards come compliant (USDA-FSIS, 2015b). The new and revised performance standards are designed to achieve the Healthy People 2020 goal of 33 % reduction in *Campylobacter-*related illnesses attributed to poultry products.

The main objective of the survey was to document the status of post-harvest *Campylobacter* control programs after the USDA-FSIS pause in enforcement. USDA-FSIS and an establishment’s internal monitoring programs were compared to determine if the company’s data can serve as a reliable predictor of performance. In addition, carcass and cut-up parts were compared to determine if the carcass performance standard can serve as a predictor of parts performance standard results. Current intervention use was collected to document *Campylobacter* specific controls used by the poultry industry to meet USDA-FSIS reduction goals.

## Materials and methods

### Survey

A cross-sectional study was conducted between March and April 2022, following the methods described by Hwang and Singer (2020). The survey targeted key stakeholders in the U.S. broiler and turkey industry using a web-based survey software (Qualtrics, Provo, UT). Stakeholders included broiler and turkey processing plants dedicated to producing ready-to-cook (RTC) parts.

The survey was divided into three sections: the first section focused on collecting basic information about the processing facility. The food safety managers within the poultry processing establishments were asked to record data on production capacity such as the number of birds slaughtered per week and average live bird weight. Participants were also asked to record data on outputs, such as the average daily volume in pounds of whole carcasses and parts for the 2021 calendar year. Average daily volume ranges were obtained from the USDA-FSIS Public Health Information System (PHIS; FSIS, 2016).

In the second section, respondents were asked to provide information on whole carcasses and parts *Campylobacter* performance standards results and prevalence for 2021. Respondents were asked to provide the facility’s *Campylobacter* performance standards results from the USDA-FSIS and from the establishment’s own testing program (internal establishment sampling). The results were compared to observe correlations between both types of samples to determine if the internal company sampling serves as a predictor of performance. The last section of the survey focused on post-chill mitigation strategies adopted by each establishment. Respondents were asked to record the antimicrobial interventions used and application methods at second processing.

The questionnaire was reviewed by the National Chicken Council and industry food safety professionals. Fresh poultry processing members of the U.S. Poultry & Egg Association were invited to participate, and the online survey was distributed to all the poultry processing facilities of the members that accepted the invitation. Expert elicitation was used to gather additional information on *Campylobacter* microbiological verification testing methods. Participation was voluntary, uncompensated, and anonymous, with no personal or company-specific data collected.

### Statistical analysis

Responses represented poultry processing establishments producing Not-Ready-to-Eat (NRTE) raw intact, and raw non-intact poultry. Data were presented as percentages or responses of each category. Chi-squared tests and Fisher’s exact tests were used to compare the association of production parameters on *Campylobacter* performance standards results and prevalence. All statistical analyses were performed using JMP software (*JMP® Pro 17*, 2023)

## Results

A total of 62 responses were collected from broiler establishments, representing 30 % of registered facilities handling raw intact, and raw non-intact poultry (USDA-FSIS, 2023). These establishments account for 30 % of weekly chicken slaughter volumes and average live bird weights compared to the National Agricultural Statistical Service (NASS, 2022). A majority of respondents (62 %) reported a company-led testing program for *Campylobacter,* while 74 % indicated an active *Campylobacter* control program for cut-up parts. Two responses were obtained from establishments producing comminuted chicken. Both indicated that the establishment was in category 1 status. A total of 10 responses were from turkey processing plants accounting for 20 % of total RTC establishments in the U.S. (Data not shown) (USDA-FSIS, 2023). All turkey facilities fell in category 1 and had prevalences of less than 10 % on carcasses and parts from USDA-FSIS and company sampling. Comminuted chicken and turkey establishment responses were not included in the overall analysis and only broiler carcass and parts data was analyzed.

### Campylobacter performance standards

USDA-FSIS categorizes establishments into three performance categories based on a 52-week sampling window for carcasses and parts (Table 1). Establishments in Categories 1 and 2 pass the performance standard and those in Category 3 fail the standard. *Campylobacter* category responses for carcasses and parts from USDA-FSIS and from internal establishment samples were analyzed to observe if an internal establishment sampling program can serve as a predictor of compliance with the USDA-FSIS performance standard for carcasses and parts. Survey responses indicated that 40 % and 22 % of establishments were in Categories 1 and 2, respectively based on USDA-FSIS carcass samples, whereas internal establishment responses placed 60 % and 11 % in these categories. For parts, 38 % and 11 % of responses met Categories 1 and 2 respectively via USDA-FSIS parts samples, compared to 56 % and 13 % via internal establishment samples (Table 2). The majority of establishments passed the performance standard for carcasses and parts, but there is a decrease in compliance with chicken parts when compared to carcasses.

**Table 1:** Current and Proposed USDA-FSIS *Campylobacter* Performance Standards.

**(A) *Campylobacter* Performance Standard**		
Product	Maximum Acceptable %Positive	Performance Standard
Broiler Carcasses	15.7 %	8/51
Chicken Parts	7.7 %	4/52
Comminuted Chicken	9.6 %	5/52
		
		
**(B) *Campylobacter* Performance Standard Category Definitions**		
Category	Definition	
1	Establishments that have achieved 50 percent or less of the maximum allowable percent positive during the most recently completed 52-week moving window.	
2	Establishments that meet the maximum allowable percent positive but have results greater than 50 percent of the maximum allowable percent positive during the most recently completed 52-week moving window.	
3	Establishments that have exceeded the maximum allowable percent positive during the most recently completed 52-week moving window.

**Table 2:** *Campylobacter* Performance Standard Responses.

				
USDA FSIS Category	USDA-FSIS Carcass Sampling	Establishment Carcass Sampling	USDA-FSIS Parts Sampling	Establishment Parts Sampling
Category 1	40 %	60 %	38 %	56 %
Category 2	22 %	11 %	11 %	13 %
Category 3	19 %	3 %	29 %	8 %
NA	19 %	25 %	22 %	24 %

Total, Responses (*n* = 62).

NA = No response.

Carcass data was used to determine if it serves as a predictor of parts performance. A significant association (*P* ≤ 0.0001) existed between whole carcass and chicken parts performance standards categories (Table 3 A-B). Establishments meeting carcasses standards were likely to meet parts standards. Notably, 92 % of responses of Category 1 carcasses from USDA-FSIS samples aligned with Category 1 parts, while Category 2 carcasses often led to Category 3 parts, suggesting cross contamination during processing. Internal testing mirrored this trend but showed Category 2 carcasses yielding Category 1 parts (33 %), potentially due to sampling variability.

**Table 3:** Association Between Chicken Carcass *Campylobacter* Performance Standards and Parts Performance Standards.

(A) USDA-FSIS Performance Standard Samples			
	USDA-FSIS Parts Performance Standard		
USDA-FSIS Carcass Performance Standard	Category 1	Category 2	Category 3
Category 1	92%	8%	0%
Category 2	8%	33%	58%
Category 3	0%	9%	91%
n= 47, χ²= 41.485, DF= 4, P-Value ≤ 0.0001			
			
(B) Establishment Performance Standard Samples			
	Establishment Parts Performance Standard		
Establishment Carcass Performance Standard	Category 1	Category 2	Category 3
Category 1	81%	11%	8%
Category 2	33%	67%	0%
Category 3	0%	0%	100%
n= 45, χ²= 27.765, DF= 4, P-Value ≤ 0.0001			
			
(C) Carcass *Campylobacter* Performance Standard			
	Establishment Carcass Performance Standard		
USDA-FSIS Carcass Performance Standard	Category 1	Category 2	Category 3
Category 1	95%	5%	0%
Category 2	58%	33%	8%
Category 3	75%	17%	8%
n= 46, χ²=7.474, DF= 4, P-Value ≤ 0.0406			
			
(D) Parts *Campylobacter* Performance Standard			
	Establishment Parts Performance Standard		
USDA-FSIS Parts Performance Standard	Category 1	Category 2	Category 3
Category 1	91%	9%	0%
Category 2	50%	50%	0%
Category 3	56%	17%	28%

n= 46, χ²= 14.503, DF= 4, P-Value ≤ 0.0058

Tables 4C showed a weak but positive correlation (*P* ≤ 0.0406 and *P* ≤ 0.0058) between USDA-FSIS and internal establishment carcass results. Establishments passing USDA-FSIS standards (Category 1 and 2) typically passed the performance standard using internal establishment samples, though establishments in Category 3 with USDA-FSIS samples occasionally corresponded with the same category using internal establishment samples (8 %).

**Table 4:** *Campylobacter* Prevalence Responses.

Campylobacter prevalence (%)	USDA FSIS Carcass Sampling	Establishment Carcass Sampling	USDA FSIS Parts Sampling	Establishment Parts Sampling
0	2 %	24 %	3 %	21 %
1-10	32 %	40 %	35 %	39 %
11-20	16 %	2 %	19 %	6 %
21-30	14 %	3 %	8 %	5 %
31-40	2 %	2 %	2 %	2 %
41-50	2 %	3 %	3 %	5 %
>50	6 %	2 %	5 %	0 %
NA	27 %	24 %	25 %	23 %

Total, Responses (*n* = 62).

NA = No Response.

Tables 4D showed a stronger positive correlation (*P* ≤ 0.0058) between USDA-FSIS and internal establishment parts results. Establishments passing USDA-FSIS standards (Category 1 and 2) typically passed the performance standard using internal establishment samples, though establishments in Category 3 with USDA-FSIS samples more frequently corresponded with the same category using internal establishment samples (28 %).

The correlations indicate that internal establishment results can provide a snapshot of the effectiveness of the control program therefore allowing the establishment to apply improvements on a consistent basis and improve its *Campylobacter* reduction capabilities. Production metrics (slaughter volume, bird weight, daily output) showed no correlations (overall *P* ≥ 0.05) with performance outcomes (supplementary Tables 1-3). Production metrics do not influence carcass or parts performance standard results.

### Campylobacter prevalence

A total of 73 % of establishments (*n* = 45) provided *Campylobacter* prevalence results from USDA-FSIS carcass sampling. 76 % (*n* = 47) of establishments provided results for internal company carcass sampling. 75 % (*n* = 46) of establishments provided results from USDA-FSIS prevalence results for parts. 77 % (*n* = 47) of establishments provided results from establishment sampling for parts (Table 4).

Most establishments reported low prevalence (< 20 %) based on USDA-FSIS (50 %) and internal establishment sampling (66 %) for carcasses. As the USDA-FSIS *Campylobacter* performance standard for whole carcasses is 15.7 %, establishments that reported prevalence below 20 % are likely to meet the *Campylobacter* performance standard for whole carcasses. For chicken parts, 38 % (USDA-FSIS) and 60 % (internal establishment) sampling fell below 10 % (Table 4). As the USDA-FSIS *Campylobacter* performance standard for parts is 7.7 %, establishments that reported prevalences below 10 % likely met the *Campylobacter* performance standard for parts.

Chi-square tests of independence were performed to examine the relation between whole carcass prevalence and chicken parts prevalence (Table 5 A- B). A correlation (*P* ≤ 0.0001) between whole carcass and chicken parts prevalence was observed, based on both the USDA-FSIS and internal establishment responses. This correlation shows that low carcass prevalence (≤ 20 %) carries over to parts where results below 10 % will result in category 1 status. Higher parts prevalence is related to higher carcass prevalence. *Campylobacter* prevalence on whole carcasses serves as an indicator of *Campylobacter* prevalence for chicken parts. Additionally, a correlation (*P* ≤ 0.0067 and *P* ≤ 0.0317) between USDA-FSIS and establishment prevalence was observed, based on both whole carcass and parts sampling (Table 5 C- D). This result show that it is likely to obtain low carcass and parts prevalence (≤ 20 % or 10 %) from the USDA-FSIS sampling schedule if the internal establishment sampling results in low prevalence.

**Table 5:** Association Between Chicken Carcass and Parts *Campylobacter* Prevalence.

(A) USDA-FSIS Sampling Prevalence																						
	USDA-FSIS Parts Prevalence																					
USDA-FSIS Carcass Prevalence	0%		1-10%			11-20%			21-30%			31-40%			41-50%				>50%			
0%	0%		100%			0%			0%			0%			0%				0%			
1-10%	11%		89%			0%			0%			0%			0%				0%			
11-20%	0%		20%			70%			10%			0%			0%				0%			
21-30%	0%		0%			38%			38%			13%			0%				13%			
31-40%	0%		0%			0%			0%			0%			0%				100%			
41-50%	0%		0%			0%			0%			0%			100%				0%			
>50%	0%		25%			25%			0%			0%			25%				25%			
n= 44, χ²= 87.70, DF= 36, P-Value ≤ 0.0001																						
(B) Establishment Sampling Prevalence																						
	Establishment Parts Prevalence																					
Establishment Carcass Prevalence	0%		1-10%			11-20%			21-30%			31-40%			41-50%				>50%			
0%	80%		20%			0%			0%			0%			0%				0%			
1-10%	4%		75%			13%			8%			0%			0%				0%			
11-20%	0%		100%			0%			0%			0%			0%				0%			
21-30%	0%		0%			0%			50%			50%			0%				0%			
31-40%	0%		0%			0%			0%			0%			100%				0%			
41-50%	0%		0%			0%			0%			0%			100%				0%			
>50%	0%		0%			100%			0%			0%			0%				0%			
n= 46, χ²= 114.724, DF= 30, P-Value ≤ 0.0001																						
(C) Carcass Sampling Prevalence																						
		Establishment Carcass Prevalence																				
USDA-FSIS Carcass Prevalence		0%			1-10%			11-20%			21-30%			31-40%			41-50%			>50%		
0%		100%			0%			0%			0%			0%			0%			0%		
1-10%		28%			72%			0%			0%			0%			0%			0%		
11-20%		40%			50%			10%			0%			0%			0%			0%		
21-30%		22%			56%			0%			11%			11%			0%			0%		
31-40%		0%			0%			0%			100%			0%			0%			0%		
41-50%		0%			100%			0%			0%			0%			0%			0%		
>50%		25%			0%			0%			0%			0%			50%			25%		
n= 44, χ²= 67.307, DF= 36, P-Value ≤ 0.0067																						
(D) Parts Sampling Prevalence																						
				Establishment Parts Prevalence																		
USDA-FSIS Parts Prevalence				0%			1-10%			11-20%			21-30%			31-40%		41-50%			>50%	
0%				100%			0%			0%			0%			0%		0%			0%	
1-10%				19%			71%			5%			0%			0%		5%			0%	
11-20%				42%			25%			17%			17%			0%		0%			0%	
21-30%				20%			60%			0%			20%			0%		0%			0%	
31-40%				0%			100%			0%			0%			0%		0%			0%	
41-50%				0%			50%			0%			0%			0%		50%			0%	
>50%				33%			0%			0%			0%			33%		33%			0%	

n= 45, χ²= 42.235, DF= 30, P-Value ≤ 0.0317

Internal establishment testing is performed mostly for verification and correlation purposes. USDA-FSIS prevalence (>20 %) correlated with elevated internal results, though outliers suggested protocol or methodological discrepancies (e.g., shipping delays, detection methods). However, no correlation (overall *P* ≥ 0.05) was observed between production metrics (slaughter volume, bird weight, daily output) and Campylobacter prevalence (Supplementary Tables 4-6).

### Campylobacter controls

Respondents were asked to identify all antimicrobials used in the processing operation, including PAA, chlorine as sodium hypochlorite and measured as free available chlorine, CPC, TSP, ASC, ClO_2_, and ultraviolet light (UV). The majority (90 %) of the establishments used PAA as the main antimicrobial intervention (Table 6), applied via combination of dip tanks and spray cabinets (60.7 %). Chlorine is being used by 50 % of the establishments, but mostly in spray cabinets (30 %) or in a combination of spray cabinets and on conveyor belts (18.2 %). A few establishments (9 %) use chlorine only on belts and fewer (3 %) use chlorine in a combination of dip tanks, spray cabinets, and conveyor belts. 3.2 % of establishments use CPC and it is applied using spray cabinets. There was no reported use of ASC, TSP, chlorine dioxide, ozone, or UV.

**Table 6:** *Campylobacter* Interventions for Post-chill Cut-up Parts.

	
	% of Use (n= 62)
Intervention	
Peroxyacetic Acid	90.3
	
Application Method	
Dip Tank only	8.9
Dip Tank and Spray Cabinet	60.7
Dip Tank and Belts	7.1
Spray Cabinets and Belts	1.8
Dip Tank, Spray Cabinets, and Belts	19.6
	
Intervention	
Chlorine (sodium hypochlorite)- Measured as Free Available Chlorine	50
	
Application Method	
Spray Cabinet only	30.3
Belts only	9
Spray Cabinets and Belts	18.2
Dip Tank, Spray Cabinets, and Belts	3
	
Intervention	
Cetylpyridinium chloride (CPC)	3.2
	
Application Method	
Spray Cabinet only	100

PAA is the most commonly used antimicrobial (90 %) in the chicken processing establishments among participants. A correlation between the type of antimicrobial used (PAA or chlorine) and *Campylobacter* prevalence for whole carcasses or chicken parts based on USDA-FSIS sampling or the internal establishment sampling (*P* ≥ 0.05) was not observed (data not shown). The responses collected from *Campylobacter* interventions were insufficient to establish a relationship between antimicrobial use and prevalence.

## Discussion

This survey evaluated the preparedness of U.S. poultry processors in meeting *Campylobacter* performance standards for chicken parts, with focus on the interplay between whole carcass and parts testing. The survey also observed whether the industry continued its *Campylobacter* control program after a pause in regulatory enforcement of performance standards. The overall findings revealed that most establishments monitor *Campylobacter* prevalence in addition to ongoing USDA-FSIS sampling for *Campylobacter* as part of their control program and implemented control measures.

While USDA-FSIS (2015a) projected that 52 % of establishments be compliant with chicken parts standard upon implementation. The survey responses indicated that the industry exceeded the risk assessment’s estimates using the internal establishment microbial verification testing. *Campylobacter* prevalence was low, with a majority of responses (60 %) indicating *Campylobacter* prevalence ≤ 10 % of chicken parts samples. The industry does not meet the risk assessment’s estimates using the USDA-FSIS microbial verification testing. 38 % of responses indicated *Campylobacter* prevalence ≤ 10 % of chicken parts samples. Results from the *Campylobacter* performance standard and prevalence results trended lower on establishment samples compared to USDA-FSIS sampling.

Establishments meeting whole carcass standards were more likely to comply with parts standards, underscoring the correlation between carcass and parts prevalence. Notably, production metrics (number of birds slaughtered per week, average live bird weight, and average daily volume) did not significantly influence *Campylobacter* prevalence, suggesting that operational scale does not compromise control efficacy. This consistency likely stems from process enhancement since 2015 standards were introduced.

The differences between USDA-FSIS and internal establishment testing results (prevalence) were discussed with industry food safety experts. The main cause might be that poultry processing establishments may collect samples more frequently than USDA-FSIS as part of their food safety program verification activities. USDA-FSIS collects a sample based on a schedule and the frequency may vary depending on the size of the establishment. Processing establishments may have a larger sample size allowing for increased precision. *Campylobacter* recovery may be dependent on the microbiological method employed. Some establishments utilize the same protocols as the USDA-FSIS, while others may utilize PCR or different types of enrichment and culture methods. Some processing establishments may have a diagnostic laboratory on site; some may have a corporate laboratory where samples can be shipped or may need to send samples to an accredited off-site laboratory. Shipping can affect *Campylobacter* recovery if there are delivery delays, temperature deviations, or sample contamination in route. Williams et al. (2010) observed different recovery rates using different carcass rinse volumes. There is also evidence that the rinse type and the type of enrichment and plating method have different sensitivities that may influence concentration and prevalence data from studies (Gonsalves et al., 2016; Hiett, 2017; Line et al., 2001). Consistency in sampling, detection protocols, and correlation between the agency and the establishments are needed to get reliable data.

Post-chill interventions in chicken parts revealed widespread adoption of peroxyacetic acid (90.3 %), primarily applied via immersion treatment (60.7 %) to maximize antimicrobial contact (Gonzalez et al., 2021). Survey responses did not establish a relation between *Campylobacter* prevalence and PAA use in chicken parts. PAA use has increased after the implementation of the *Salmonella* parts performance standards in parts and comminuted poultry. Studies have shown that PAA is more effective at reducing *Campylobacter* compared to chlorine (Chen et al., 2014; Laranja et al., 2023; Zhang et al., 2018). Its increased use over chlorine may be due to PAA’s effect not being diminished by the organic matter present in tank water and surfaces while chlorine’s effectiveness is diminished by the presence of organic matter, thus retaining its effectiveness over time (Zhang et al., 2018).

Survey responses indicated that 50 % of establishments use chlorine as an antimicrobial, but its application is mostly by spray (30.3 %), or in combination with application on belts (18.2 %). The survey could not establish a relationship between *Campylobacter* and chlorine use in chicken parts. CPC use in the establishments was low (3.2 %) although it is also effective at reducing *Campylobacter* but additional equipment is needed to recover and neutralize the antimicrobial due to carryover effects in the process downstream, making its adoption costly (Zhang et al., 2018).

Many of the antimicrobials that are used for meeting the *Campylobacter* performance standards are similar to those used in *Salmonella* control programs and processing establishments (Zhang et al., 2018). All survey responses indicated that there is a *Salmonella* control program in conjunction with the *Campylobacter* control program. Current interventions allow most processing establishments to meet the *Campylobacter* performance standards for chicken parts, but *Campylobacter* specific interventions need to be evaluated separately from *Salmonella* control interventions.

The survey presented several limitations. The survey took longer than the estimated 30 minutes, causing incomplete responses and variable testing protocols limited analysis. A future survey should standardize data collection (e.g., seasonal prevalence, sample counts, sampling, and plating methods) and evaluate preharvest interventions to reduce Campylobacter load pre-processing.

In conclusion, since the 2015 implementation of performance standards, the U.S. broiler industry has reduced *Campylobacter* prevalence through antimicrobial interventions and enhanced monitoring. The internal sampling programs serve as an indicator of USDA-FSIS performance standard results. Carcass prevalence influences parts prevalence. However, disparities in USDA-FSIS compliance highlight the need for refined protocols, consistent methodologies, and integrated preharvest strategies. Addressing these gaps will be pivotal in achieving public health objectives and ensuring sustained improvements in poultry safety.

## CRediT authorship contribution statement

**Rafael E. Rivera:** Writing – review & editing, Writing – original draft, Visualization, Validation, Supervision, Software, Resources, Project administration, Methodology, Investigation, Funding acquisition, Formal analysis, Data curation, Conceptualization. **Harshavardhan Thippareddi:** Writing – review & editing, Supervision, Methodology, Conceptualization. **Sanjay Kumar:** Writing – review & editing, Visualization. **Manpreet Singh:** Writing – review & editing, Validation, Methodology, Conceptualization.

## Disclosures

The authors declare the following financial interests/personal relationships which may be considered as potential competing interests:

Rafael E. Rivera Betancourt reports administrative support was provided by US Poultry and Egg Association. If there are other authors, they declare that they have no known competing financial interests or personal relationships that could have appeared to influence the work reported in this paper.

## Appendix Supplementary materials

Image, application 1

Image, application 2

## Footnotes

Supplementary material associated with this article can be found, in the online version, at doi:10.1016/j.psj.2025.105924.
