# Supplementary material for: Survey of U.S. broiler establishments on Campylobacter Performance standards for parts
Source: Poult Sci. 2025 Sep 30;104(12):105924. doi: 10.1016/j.psj.2025.105924 (PMC12538697; doi:10.1016/j.psj.2025.105924)
Supplement: Supplementary file 2 [file mmc2.docx]

| **Supplementary Table 1: Association Between the Amount of Birds Slaughtered/Week and *Campylobacter* Performance Standard** | | | | |
| --- | --- | --- | --- | --- |
|  | |  |  |  |
| (A) USDA-FSIS Carcass *Campylobacter* Performance Standard | | | | |
|  | USDA-FSIS Carcass *Campylobacter* Performance Standard | | | |
| Birds Slaughtered/Week | Category 1 | | Category 2 | Category 3 |
| 0-250000 | 60% | | 40% | 0% |
| 25001-500000 | 67% | | 33% | 0% |
| 500001-750000 | 43% | | 29% | 29% |
| 750001-1000000 | 55% | | 27% | 18% |
| 1000001-1250000 | 31% | | 25% | 44% |
| 1250001-1500000 | 71% | | 14% | 14% |
| 1500501-1750000 | 100% | | 0% | 0% |
| n= 50, χ²= 9.002, DF= 12, P-Value ≤ 0.7857 | | | |  |
|  | |  |  |  |
| (B) Establishment Carcass *Campylobacter* Performance Standard | | | | |
|  | Establishment Carcass *Campylobacter* Performance Standard | | | |
| Birds Slaughtered/Week | | Category 1 | Category 2 | Category 3 |
| 0-250000 | | 75% | 25% | 0% |
| 25001-500000 | | 75% | 0% | 25% |
| 500001-750000 | | 86% | 14% | 0% |
| 750001-1000000 | | 73% | 18% | 9% |
| 1000001-1250000 | | 92% | 8% | 0% |
| 1250001-1500000 | | 71% | 29% | 0% |
| 1500501-1750000 | | 100% | 0% | 0% |
| n= 47, χ²= 8.921, DF= 12, P-Value ≤ 0.7237 | | | |  |
|  | |  |  |  |
| (C) USDA-FSIS Parts *Campylobacter* Performance Standard | | | | |
|  | | USDA-FSIS Parts *Campylobacter* Performance Standard | | |
| Birds Slaughtered/Week | | Category 1 | Category 2 | Category 3 |
| 0-250000 | | 57% | 14% | 29% |
| 25001-500000 | | 67% | 0% | 33% |
| 500001-750000 | | 43% | 14% | 43% |
| 750001-1000000 | | 44% | 33% | 22% |
| 1000001-1250000 | | 33% | 13% | 53% |
| 1250001-1500000 | | 71% | 0% | 29% |
| 1500501-1750000 | | 100% | 0% | 0% |
| n= 49, χ²= 8.590, DF= 12, P-Value ≤ 0.8307 | | | |  |
|  | |  |  |  |
| **Supplementary Table 1 (Continued): Association Between the Amount of Birds Slaughtered/Week and *Campylobacter* Performance Standard**  (D) Establishment Parts *Campylobacter* Performance Standard | | | | |
|  | | Establishment Parts *Campylobacter* Performance Standard | | |
| Birds Slaughtered/Week | | Category 1 | Category 2 | Category 3 |
| 0-250000 | | 67% | 33% | 0% |
| 25001-500000 | | 75% | 0% | 25% |
| 500001-750000 | | 57% | 14% | 29% |
| 750001-1000000 | | 56% | 33% | 11% |
| 1000001-1250000 | | 93% | 0% | 7% |
| 1250001-1500000 | | 71% | 29% | 0% |
| 1500501-1750000 | | 100% | 0% | 0% |
| n= 48, χ²= 12.435, DF= 12, P-Value ≤ 0.2407 | | | |  |

| **Supplementary Table 2: Association Between Average Live Bird Weight in Pounds (LBS) and *Campylobacter* Performance Standard** | | | |
| --- | --- | --- | --- |
| (A) USDA-FSIS Carcass *Campylobacter* Performance Standard | | | |
|  | USDA-FSIS Carcass *Campylobacter* Performance Standard | | |
| Live Bird Weight | Category 1 | Category 2 | Category 3 |
| < 5 LBS | 45% | 18% | 36% |
| 5-7 LBS | 44% | 38% | 19% |
| 7-9 LBS | 45% | 9% | 45% |
| > 9 LBS | 60% | 40% | 0% |
| n= 48, χ²= 8.443, DF= 6, P-Value ≤ 0.1938 | |  |  |
| (B) Establishment Carcass *Campylobacter* Performance Standard | | | |
|  | Establishment Carcass *Campylobacter* Performance Standard | | |
| Live Bird Weight | Category 1 | Category 2 | Category 3 |
| < 5 LBS | 80% | 20% | 0% |
| 5-7 LBS | 67% | 27% | 7% |
| 7-9 LBS | 100% | 0% | 0% |
| > 9 LBS | 80% | 10% | 10% |
| n= 45, χ²= 5.655, DF= 6, P-Value ≤ 0.4716 | |  |  |
| (C) USDA-FSIS Parts *Campylobacter* Performance Standard | | | |
|  | USDA-FSIS Carcass *Campylobacter* Performance Standard | | |
| Live Bird Weight | Category 1 | Category 2 | Category 3 |
| < 5 LBS | 44% | 0% | 56% |
| 5-7 LBS | 47% | 20% | 33% |
| 7-9 LBS | 45% | 18% | 36% |
| > 9 LBS | 60% | 10% | 30% |
| n= 45, χ²= 3.305, DF= 6, P-Value ≤ 0.8467 | |  |  |
| (D) Establishment Parts *Campylobacter* Performance Standard | | | |
|  | Establishment Carcass *Campylobacter* Performance Standard | | |
| Live Bird Weight | Category 1 | Category 2 | Category 3 |
| < 5 LBS | 80% | 20% | 0% |
| 5-7 LBS | 71% | 14% | 14% |
| 7-9 LBS | 70% | 10% | 20% |
| > 9 LBS | 70% | 20% | 10% |
| n= 44, χ²= 2.464, DF= 6, P-Value ≤ 0.9173 | |  |  |

| **Supplementary Table 3: Association Between WOG and Parts Average Daily Volume in Pounds (LBS) and *Campylobacter* Performance Standard** | | | |
| --- | --- | --- | --- |
| (A) USDA-FSIS Carcass *Campylobacter* Performance Standard | | | |
|  | USDA-FSIS Carcass *Campylobacter* Performance Standard | | |
| LBS. WOG/Day | Category 1 | Category 2 | Category 3 |
| 1001-3000 LBS | 25% | 50% | 25% |
| 50001-250000 LBS | 31% | 31% | 38% |
| 250001-600000 LBS | 86% | 0% | 14% |
| 600001-1000000 LBS | 43% | 29% | 29% |
| >1000000 LBS | 33% | 33% | 33% |
| n= 37, χ²= 7.621, DF= 8, P-Value ≤ 0.5140 | | |  |
| (B) Establishment Carcass *Campylobacter* Performance Standard | | | |
|  | Establishment Carcass *Campylobacter* Performance Standard | | |
| LBS. WOG/Day | Category 1 | Category 2 | Category 3 |
| 1001-3000 LBS | 75% | 25% | 0% |
| 50001-250000 LBS | 77% | 23% | 0% |
| 250001-600000 LBS | 100% | 0% | 0% |
| 600001-1000000 LBS | 71% | 29% | 0% |
| >1000000 LBS | 83% | 0% | 17% |
| n= 38, χ²= 9.400, DF= 8, P-Value ≤ 0.3067 | | |  |
| (C) USDA-FSIS Chicken Parts *Campylobacter* Performance Standard | | | |
|  | USDA-FSIS Parts *Campylobacter* Performance Standard | | |
| LBS. Parts/Day | Category 1 | Category 2 | Category 3 |
| 6001-50000 LBS | 100% | 0% | 0% |
| 50001-250000 LBS | 50% | 13% | 38% |
| 250001-600000 LBS | 50% | 17% | 33% |
| 600001-1000000 LBS | 11% | 22% | 67% |
| >1000000 LBS | 78% | 0% | 22% |
| n= 45, χ²= 9.762, DF= 8, P-Value ≤ 0.1931 | | |  |
| (D) Establishment Chicken Parts *Campylobacter* Performance Standard | | | |
|  | Establishment Parts *Campylobacter* Performance Standard | | |
| LBS. WOG/Day | Category 1 | Category 2 | Category 3 |
| 6001-50000 LBS | 100% | 0% | 0% |
| 50001-250000 LBS | 75% | 13% | 13% |
| 250001-600000 LBS | 89% | 11% | 0% |
| 600001-1000000 LBS | 33% | 33% | 33% |
| >1000000 LBS | 75% | 13% | 13% |
| n= 45, χ²= 11.472, DF= 8, P-Value ≤ 0.1058 | | |  |

| **Supplementary Table 4: Association Between the Amount of Birds Slaughtered/Week and *Campylobacter* Prevalence** | | | | | | | | | | | | | | |
| --- | --- | --- | --- | --- | --- | --- | --- | --- | --- | --- | --- | --- | --- | --- |
| (A) USDA-FSIS Carcass *Campylobacter* Prevalence | | | | | | | | |  | |  | |  | |
|  | | USDA-FSIS Carcass *Campylobacter* Prevalence | | | | | | | | | | |  | |
| Birds Slaughtered/Week | | 0% | 1-10% | | 11-20% | | 21-30% | | 31-40% | | 41-50% | | >50% | |
| 0-250000 | | 0% | 100% | | 0% | | 0% | | 0% | | 0% | | 0% | |
| 25001-500000 | | 0% | 67% | | 33% | | 0% | | 0% | | 0% | | 0% | |
| 500001-750000 | | 0% | 29% | | 14% | | 43% | | 0% | | 0% | | 14% | |
| 750001-1000000 | | 0% | 55% | | 9% | | 18% | | 9% | | 0% | | 9% | |
| 1000001-1250000 | | 0% | 15% | | 54% | | 15% | | 0% | | 8% | | 8% | |
| 1250001-1500000 | | 0% | 67% | | 0% | | 17% | | 0% | | 0% | | 17% | |
| 1500501-1750000 | | 50% | 0% | | 0% | | 50% | | 0% | | 0% | | 0% | |
| n= 46, χ²= 51.516, DF= 36, P-Value ≤ 0.1084 | | | | |  | |  | |  | |  | |  | |
| (B) Establishment Carcass *Campylobacter* Prevalence | | | | | | | | |  | |  | |  | |
|  | | Establishment Carcass *Campylobacter* Prevalence | | | | | | | | | | |  | |
| Birds Slaughtered/Week | | 0% | 1-10% | | 11-20% | | 21-30% | | 31-40% | | 41-50% | | >50% | |
| 0-250000 | | 0% | 100% | | 0% | | 0% | | 0% | | 0% | | 0% | |
| 25001-500000 | | 50% | 25% | | 25% | | 0% | | 0% | | 0% | | 0% | |
| 500001-750000 | | 14% | 71% | | 0% | | 0% | | 0% | | 0% | | 14% | |
| 750001-1000000 | | 27% | 55% | | 0% | | 9% | | 0% | | 9% | | 0% | |
| 1000001-1250000 | | 46% | 54% | | 0% | | 0% | | 0% | | 0% | | 0% | |
| 1250001-1500000 | | 33% | 33% | | 0% | | 0% | | 17% | | 17% | | 0% | |
| 1500501-1750000 | | 50% | 0% | | 0% | | 50% | | 0% | | 0% | | 0% | |
| n= 47, χ²= 46.911, DF= 36, P-Value ≤ 0.1621 | | | | |  | |  | |  | |  | |  | |
| (C) USDA-FSIS Parts *Campylobacter* Prevalence | | | | | | | | | |  | |  | |  |
|  | USDA-FSIS Parts *Campylobacter* Prevalence | | | | | | | | | | | | |  |
| Birds Slaughtered/Week | 0% | | | 1-10% | | 11-20% | | 21-30% | | 31-40% | | 41-50% | | >50% |
| 0-250000 | 0% | | | 83% | | 17% | | 0% | | 0% | | 0% | | 0% |
| 25001-500000 | 0% | | | 75% | | 0% | | 25% | | 0% | | 0% | | 0% |
| 500001-750000 | 0% | | | 29% | | 57% | | 14% | | 0% | | 0% | | 0% |
| 750001-1000000 | 10% | | | 50% | | 20% | | 0% | | 10% | | 0% | | 10% |
| 1000001-1250000 | 8% | | | 17% | | 42% | | 17% | | 0% | | 8% | | 8% |
| 1250001-1500000 | 0% | | | 67% | | 0% | | 0% | | 0% | | 17% | | 17% |
| 1500501-1750000 | 0% | | | 50% | | 0% | | 50% | | 0% | | 0% | | 0% |
| n= 47, χ²= 31.972, DF= 36, P-Value ≤ 0.3545 | | | | | |  | |  | |  | |  | |  |

**Supplementary Table 4 (Continued): Association Between the Amount of Birds Slaughtered/Week and *Campylobacter* Prevalence**

| (D) Establishment Parts *Campylobacter* Prevalence | | | | |  |  |  |
| --- | --- | --- | --- | --- | --- | --- | --- |
|  | Establishment Parts *Campylobacter* Prevalence | | | | | |  |
| Birds Slaughtered/Week | 0% | 1-10% | 11-20% | 21-30% | 31-40% | 41-50% | >50% |
| 0-250000 | 0% | 100% | 0% | 0% | 0% | 0% | 0% |
| 25001-500000 | 25% | 75% | 0% | 0% | 0% | 0% | 0% |
| 500001-750000 | 14% | 43% | 14% | 29% | 0% | 0% | 0% |
| 750001-1000000 | 30% | 40% | 10% | 0% | 10% | 10% | 0% |
| 1000001-1250000 | 46% | 38% | 15% | 0% | 0% | 0% | 0% |
| 1250001-1500000 | 33% | 33% | 0% | 0% | 0% | 33% | 0% |
| 1500501-1750000 | 0% | 50% | 0% | 50% | 0% | 0% | 0% |
| n= 47, χ²= 46.911, DF= 36, P-Value ≤ 0.1621 | | |  |  |  |  |  |

| **Supplementary Table 5: Association Between Average Live Bird Weight in Pounds (LBS) and *Campylobacter* Prevalence** | | | | | | | | | | | | | | |
| --- | --- | --- | --- | --- | --- | --- | --- | --- | --- | --- | --- | --- | --- | --- |
| (A) USDA-FSIS Carcass *Campylobacter* Prevalence | | | | | | | | | | | | |  | |
|  | | USDA-FSIS Chicken Carcass *Campylobacter* Prevalence | | | | | | | | | | |  | |
| Live Bird Weight | | 0% | | 1-10% | | 11-20% | 21-30% | | 31-40% | | 41-50% | | >50% | |
| < 5 LBS | | 0% | | 50% | | 20% | 20% | | 0% | | 0% | | 10% | |
| 5-7 LBS | | 0% | | 29% | | 18% | 29% | | 6% | | 6% | | 12% | |
| 7-9 LBS | | 10% | | 30% | | 30% | 20% | | 0% | | 0% | | 10% | |
| > 9 LBS | | 0% | | 75% | | 25% | 0% | | 0% | | 0% | | 0% | |
| n= 45, χ²= 13.720, DF= 18, P-Value ≤ 0.8226 | | | | | | | | |  | |  | |  | |
| (B) Establishment Carcass *Campylobacter* Prevalence | | | | | | | | | | | | |  | |
|  | | Establishment Carcass *Campylobacter* Prevalence | | | | | | | | | | |  | |
| Live Bird Weight | | 0% | | 1-10% | | 11-20% | 21-30% | | 31-40% | | 41-50% | | >50% | |
| < 5 LBS | | 36% | | 45% | | 0% | 0% | | 9% | | 9% | | 0% | |
| 5-7 LBS | | 31% | | 44% | | 0% | 13% | | 0% | | 6% | | 6% | |
| 7-9 LBS | | 40% | | 60% | | 0% | 0% | | 0% | | 0% | | 0% | |
| > 9 LBS | | 22% | | 67% | | 11% | 0% | | 0% | | 0% | | 0% | |
| n= 46, χ²= 15.761, DF= 18, P-Value ≤ 0.9016 | | | | | | | | |  | |  | |  | |
| (C) USDA-FSIS Parts *Campylobacter* Prevalence | | | | | | | | | | |  | |  | |
|  | | USDA-FSIS Parts *Campylobacter* Prevalence | | | | | | | | | | |  | |
| Live Bird Weight | | 0% | | 1-10% | | 11-20% | 21-30% | | 31-40% | | 41-50% | | >50% | |
| < 5 LBS | | 11% | | 56% | | 11% | 11% | | 0% | | 0% | | 11% | |
| 5-7 LBS | | 0% | | 25% | | 38% | 13% | | 6% | | 13% | | 6% | |
| 7-9 LBS | | 0% | | 50% | | 30% | 10% | | 0% | | 0% | | 10% | |
| > 9 LBS | | 11% | | 67% | | 11% | 11% | | 0% | | 0% | | 0% | |
| n= 44, χ²= 14.287, DF= 18, P-Value ≤ 0.7482 | | | | | | | | |  | |  | |  | |
| (D) Establishment Parts *Campylobacter* Prevalence | | | | | | | | | | | |  | |  |
|  | Establishment Parts *Campylobacter* Prevalence | | | | | | | | | | | | |  |
| Live Bird Weight | 0% | | 1-10% | | 11-20% | | | 21-30% | | 31-40% | | 41-50% | | >50% |
| < 5 LBS | 36% | | 36% | | 9% | | | 0% | | 0% | | 18% | | 0% |
| 5-7 LBS | 33% | | 33% | | 7% | | | 13% | | 7% | | 7% | | 0% |
| 7-9 LBS | 20% | | 50% | | 20% | | | 10% | | 0% | | 0% | | 0% |
| > 9 LBS | 22% | | 78% | | 0% | | | 0% | | 0% | | 0% | | 0% |
| n= 45, χ²= 13.667, DF= 15, P-Value ≤ 0.6984 | | | | | | | | | |  | |  | |  |

| **Supplementary Table 6: Association Between WOG and Parts Average Daily Volume in Pounds (LBS) and *Campylobacter* Prevalence** | | | | | | | |
| --- | --- | --- | --- | --- | --- | --- | --- |
| (A) USDA-FSIS *Campylobacter* Sampling Prevalence | | | | | |  |  |
|  | USDA-FSIS *Campylobacter* Sampling Prevalence | | | | | |  |
| LBS. WOG/Day | 0% | 1-10% | 11-20% | 21-30% | 31-40% | 41-50% | >50% |
| 1001-3000 LBS | 0% | 50% | 0% | 50% | 0% | 0% | 0% |
| 50001-250000 LBS | 0% | 15% | 31% | 38% | 0% | 5% | 15% |
| 250001-600000 LBS | 0% | 83% | 0% | 0% | 0% | 0% | 17% |
| 600001-1000000 LBS | 0% | 43% | 14% | 29% | 0% | 14% | 0% |
| >1000000 LBS | 0% | 33% | 50% | 0% | 17% | 0% | 0% |
| n= 36, χ²= 26.842, DF= 20, P-Value ≤ 0.0810 | | | |  |  |  |  |
|  |  |  |  |  |  |  |  |
| (B) Establishment *Campylobacter* Sampling Prevalence | | | | | |  |  |
|  | Establishment *Campylobacter* Sampling Prevalence | | | | | |  |
| LBS. WOG/Day | 0% | 1-10% | 11-20% | 21-30% | 31-40% | 41-50% | >50% |
| 1001-3000 LBS | 0% | 75% | 0% | 0% | 25% | 0% | 0% |
| 50001-250000 LBS | 46% | 38% | 0% | 8% | 0% | 0% | 8% |
| 250001-600000 LBS | 63% | 25% | 0% | 0% | 0% | 13% | 0% |
| 600001-1000000 LBS | 14% | 86% | 0% | 0% | 0% | 0% | 0% |
| >1000000 LBS | 20% | 60% | 0% | 20% | 0% | 0% | 0% |
| n= 37, χ²= 25.216, DF= 20, P-Value ≤ 0.1438 | | | |  |  |  |  |
|  |  |  |  |  |  |  |  |
| (C) USDA-FSIS Chicken Parts *Campylobacter* Sampling Prevalence | | | | | |  |  |
|  | USDA-FSIS Parts *Campylobacter* Sampling Prevalence | | | | | | |
| LBS. Parts/Day | 0% | 1-10% | 11-20% | 21-30% | 31-40% | 41-50% | >50% |
| 6001-50000 LBS | 0% | 0% | 100% | 0% | 0% | 0% | 0% |
| 50001-250000 LBS | 0% | 30% | 30% | 40% | 0% | 0% | 0% |
| 250001-600000 LBS | 0% | 63% | 19% | 6% | 0% | 6% | 6% |
| 600001-1000000 LBS | 0% | 33% | 33% | 0% | 11% | 0% | 22% |
| >1000000 LBS | 13% | 63% | 25% | 0% | 0% | 0% | 0% |
| n= 44, χ²= 29.357, DF= 24, P-Value ≤ 0.1489 | | | |  |  |  |  |
|  |  |  |  |  |  |  |  |
| (D) Establishment Chicken Parts *Campylobacter* Sampling Prevalence | | | | | |  |  |
|  | Establishment Parts *Campylobacter* Sampling Prevalence | | | | | | |
| LBS. WOG/Day | 0% | 1-10% | 11-20% | 21-30% | 31-40% | 41-50% | >50% |
| 6001-50000 LBS | 50% | 50% | 0% | 0% | 0% | 0% | 0% |
| 50001-250000 LBS | 20% | 50% | 10% | 20% | 0% | 0% | 0% |
| 250001-600000 LBS | 35% | 53% | 6% | 0% | 0% | 6% | 0% |
| 600001-1000000 LBS | 22% | 44% | 0% | 11% | 11% | 11% | 0% |
| >1000000 LBS | 0% | 71% | 29% | 0% | 0% | 0% | 0% |
| n= 45, χ²= 18.457, DF= 20, P-Value ≤ 0.4777 | | | |  |  |  |  |
