# Supplementary material for: Survey of U.S. broiler establishments on Campylobacter Performance standards for parts
Source: Poult Sci. 2025 Sep 30;104(12):105924. doi: 10.1016/j.psj.2025.105924 (PMC12538697; doi:10.1016/j.psj.2025.105924)
Supplement: Supplementary file 2 [file mmc1.docx]

Second Processing Campylobacter Control - Final - 2

Start of Block: Q1

Q1.1 Thank you for participating in this questionnaire regarding Campylobacter in broiler and turkey production. This questionnaire is intended to gather information regarding interventions and management practices used by the U.S. broiler and turkey industry to mitigate Campylobacter contamination of parts, comminuted products, and meet proposed USDA Food Safety and Inspection Service (FSIS) performance standards. Results will help to develop recommendations in areas of opportunity for improvement.
<div> 
<div>ALL RESPONSES ARE CONFIDENTIAL AND ANONYMOUS. </div>

<div> </div>

<div>This questionnaire should take up to 30 minutes to complete. Please complete even if there is not a specific program targeting Campylobacter reduction. If you have any questions about this survey, please contact Rafael Rivera at rrivera@uspoultry.org .</div>
</div>

<div> </div>

<div><strong>TIP: HAVE MICROBIAL AND INTERVENTION DATA AVAILABLE TO SAVE ON RESPONSE TIME</strong></div>

| Page Break |  |
| --- | --- |

End of Block: Q1

Start of Block: Block 1

Q69 <span style="font-size:19px;"><strong>General Information</strong></span>
<div> </div>

<div>This section will gather preliminary data about the establishment.</div>

| Page Break |  |
| --- | --- |

Q2.2 Does your establishment process broilers, or turkeys?

- Broilers  (1) 

- Turkeys  (2) 

Q2.1 On average, how many ${q://QID3/ChoiceGroup/SelectedChoices} does your establishment slaughter per week?

________________________________________________________________

Q2.3 Which broiler live weight does your establishment slaughter?

- 0 lbs.  (5) 

- < 5 lbs.  (1) 

- 5-7 lbs.  (2) 

- 7-9 lbs.  (3) 

- > 9 lbs.  (4) 

Q2.4 Which turkey tom live weight does the establishment slaughter?

- 0 lbs.  (1) 

- < 30 lbs.  (2) 

- 30-35 lbs.  (3) 

- 35-40 lbs.  (4) 

- > 40 lbs.  (5) 

Q2.5 Which turkey hen live weight does you establishment slaughter?

- 0 lbs.  (1) 

- < 15 lbs.  (2) 

- 15-20 lbs.  (3) 

- 20-25 lbs.  (4) 

- > 25 lbs.  (5) 

Q2.6 Does your establishment include these ${q://QID3/ChoiceGroup/SelectedChoices} processing areas?    
<div> </div>

<div><span style="font-size:13px;"><strong>Please check all that apply.</strong></span></div>

- Slaughter  (1) 

- Parts cut-up  (2) 

- Deboning  (3) 

- Ground  (4) 

- Further processing  (5) 

- Other: (please specify)  (6) __________________________________________________

Q8 On average, how many pounds  of WOGs are produced per day?

- 0 lbs.  (1) 

- 1-100 lbs.  (2) 

- 101-1000 lbs.  (3) 

- 1,001-3,000 lbs.  (4) 

- 3001-6,000 lbs.  (5) 

- 6,001-50,000 lbs.  (6) 

- 50,001-250,000 lbs.  (7) 

- 250,001-6000,000 lbs.  (8) 

- 600,001-1,000,000 lbs.  (9) 

- > 1,000,000 lbs.  (10) 

Q10 On average, how many pounds  of parts are produced per day? (include deboned parts)

- 0 lbs.  (1) 

- 1-100 lbs.  (2) 

- 101-1000 lbs.  (3) 

- 1,001-3,000 lbs.  (4) 

- 3001-6,000 lbs.  (5) 

- 6,001-50,000 lbs.  (6) 

- 50,001-250,000 lbs.  (7) 

- 250,001-6000,000 lbs.  (8) 

- 600,001-1,000,000 lbs.  (9) 

- > 1,000,000 lbs.  (10) 

Q11 On average, how many pounds  of ground ${q://QID3/ChoiceGroup/SelectedChoices} are produced per day?

- 0 lbs.  (1) 

- 1-100 lbs.  (2) 

- 101-1000 lbs.  (3) 

- 1,001-3,000 lbs.  (4) 

- 3001-6,000 lbs.  (5) 

- 6,001-50,000 lbs.  (6) 

- 50,001-250,000 lbs.  (7) 

- 250,001-6000,000 lbs.  (8) 

- 600,001-1,000,000 lbs.  (9) 

- > 1,000,000 lbs.  (10) 

Q12 On average, how many pounds  of comminuted  ${q://QID3/ChoiceGroup/SelectedChoices} are produced per day?

- 0 lbs.  (1) 

- 1-100 lbs.  (2) 

- 101-1000 lbs.  (3) 

- 1,001-3,000 lbs.  (4) 

- 3001-6,000 lbs.  (5) 

- 6,001-50,000 lbs.  (6) 

- 50,001-250,000 lbs.  (7) 

- 250,001-6000,000 lbs.  (8) 

- 600,001-1,000,000 lbs.  (9) 

- > 1,000,000 lbs.  (10) 

Q13 Is any poultry sourced from an external supplier (in company and/or outside supplier) for second and further processing)?

- Yes  (1) 

- No  (2) 

Q14 Do you employ a control program for <u><strong><em>Campylobacter</em></strong></u> for parts, ground, and other comminuted poultry?

- Yes  (1) 

- No  (2) 

Q15 Do you employ a control program for <u><strong><em>Salmonella</em></strong></u> for parts, ground, and other comminuted poultry?

- Yes  (1) 

- No  (2) 

End of Block: Block 1

Start of Block: Block 2

Q16 <strong><span style="font-size:19px;">Second and Further Processing Campylobacter Control</span></strong>
<div> </div>

<div> The following questions will ask about current practices to reduce Campylobacter contamination in cut-up parts and comminuted product to meet current and proposed USDA Food Safety and Inspection Service (FSIS) performance standards. The responses will help understand the effect of microbial interventions and its effect on Campylobacter. <u>Please respond even if the establishment does not have specific Campylobacter interventions for parts. </u></div>

<div> </div>

<div>Note: There are performance standards for Campylobacter for chicken and turkey carcasses and chicken parts. The performance standards for carcass and parts were suspended after the change to Neutralizing Buffered Peptone Water for further analysis. There are proposed standards for comminuted chicken and turkey products. The performance standards and categories to be used in this survey are as follows.</div>

Q17 <table summary="*Proposed standards Currently under review." style="width:500px;" cellspacing="1" cellpadding="1" border="1" align="center">
	<caption>
	<h4 style="text-align: center;"><strong>Current and Proposed FSIS Camplylobacter Performance Standards Performance</strong></h4>
	</caption>
	<thead>
		<tr>
			<th scope="row">Product</th>
			<th scope="col">Maximum Acceptable %Positive</th>
			<th scope="col">Performance Standard</th>
		</tr>
	</thead>
	<tbody>
		<tr>
			<th scope="row">Broiler Carcasses</th>
			<td style="text-align: center;">15.7%</td>
			<td style="text-align: center;">8/51</td>
		</tr>
		<tr>
			<th scope="row">Turkey Carcasses</th>
			<td style="text-align: center;">5.4%</td>
			<td style="text-align: center;">3/56</td>
		</tr>
		<tr>
			<th scope="row">Chicken Parts</th>
			<td style="text-align: center;">7.7%</td>
			<td style="text-align: center;">4/52</td>
		</tr>
		<tr>
			<th scope="row">Comminuted Chicken*</th>
			<td style="text-align: center;">9.6%</td>
			<td style="text-align: center;">5/52</td>
		</tr>
		<tr>
			<th scope="row">Comminuted Turkey*</th>
			<td style="text-align: center;">9.6%</td>
			<td style="text-align: center;">5/52</td>
		</tr>
	</tbody>
</table>
<span style="font-size:11pt"><span style="line-height:normal"><span style="page-break-after:avoid"><span style="font-family:Calibri,sans-serif"><b><span style="font-size:12.0pt"><span style="font-family:"Times New Roman",serif">*Proposed standard currently under review</span></span></b></span></span></span></span><br>
<br>
<span style="font-size:11pt"><span style="line-height:normal"><span style="page-break-after:avoid"><span style="font-family:Calibri,sans-serif"><b><span style="font-size:12.0pt"><span style="font-family:"Times New Roman",serif">Category 1: Establishments that have achieved 50 percent or less of the maximum allowable percent positive during the most recently completed 52-week moving window.</span></span></b></span></span></span></span><br>
<span style="font-size:11pt"><span style="line-height:normal"><span style="page-break-after:avoid"><span style="font-family:Calibri,sans-serif"><b><span style="font-size:12.0pt"><span style="font-family:"Times New Roman",serif">Category 2: Establishments that meet the maximum allowable percent positive but have results greater than 50 percent of the maximum allowable percent positive during the most recently completed 52-week moving window.</span></span></b></span></span></span></span><br>
<span style="font-size:11pt"><span style="line-height:normal"><span style="page-break-after:avoid"><span style="font-family:Calibri,sans-serif"><b><span style="font-size:12.0pt"><span style="font-family:"Times New Roman",serif">Category 3: Establishments that have exceeded the maximum allowable percent positive during the most recently completed 52-week moving window.<br>
 </span></span></b></span></span></span></span><br>
 

| Page Break |  |
| --- | --- |

Q18 Does your processing establishment do its own testing for <em><strong><u>Campylobacter</u></strong></em> in second and further processing?<br>
<br>
<strong><span style="font-size:13px;">Note: Even if the response is no, continue on responding to gather information on general pathogen reduction. </span></strong>

- Yes  (1) 

- No  (2) 

Q20 Where do you take samples for <u><em><strong>Campylobacter</strong></em></u> in Second and Further Processing?
<div><strong><span style="font-size:13px;">Please check all that apply</span></strong></div>

- Cut-up  (1) 

- Deboning  (2) 

- Skinning  (3) 

- Portioning  (4) 

- Marination  (5) 

- Grinding  (6) 

- Metal detectors  (7) 

- Packaging  (8) 

- Breading  (9) 

- Other (please specify):  (10) __________________________________________________

- N/A  (11) 

Q21 Based on <u>USDA FSIS sampling</u>, what category does this plant currently fall into with respect to current and proposed performance standards for <em><u><strong>Campylobacte</strong></u>r</em>?

|  | Category 1 (1) | Category 2 (2) | Category 3 (4) | N/A (5) |
| --- | --- | --- | --- | --- |
| USDA testing carcass  (1) |  |  |  |  |
| USDA testing parts (2) |  |  |  |  |
| USDA testing comminuted (4) |  |  |  |  |

Q22 Based on <u>Company sampling</u>, what category does this plant currently falls into with respect to current and proposed performance standards for <u><em><strong>Campylobacter</strong></em></u>?

|  | Category 1 (1) | Category 2 (2) | Category 3 (3) | N/A (4) |
| --- | --- | --- | --- | --- |
| Company testing carcass (5) |  |  |  |  |
| Company testing parts (6) |  |  |  |  |
| Company testing comminuted (8) |  |  |  |  |

Q23 The current <u><em><strong>Campylobacter</strong></em></u> prevalence from <u>USDA sampling </u>is:

|  | 0 % (1) | 1-10% (2) | 11-20 % (3) | 21-30 % (4) | 31-40 % (5) | 41-50 % (6) | >50 % (7) | N/A (8) |
| --- | --- | --- | --- | --- | --- | --- | --- | --- |
| Whole carcass  (1) |  |  |  |  |  |  |  |  |
| Parts (2) |  |  |  |  |  |  |  |  |
| Comminuted (4) |  |  |  |  |  |  |  |  |

Q24 The current <u><em><strong>Campylobacter</strong></em></u> prevalence from <u>Company sampling </u>is:

|  | 0 % (1) | 1-10 % (2) | 11-20 % (3) | 21-30 % (4) | 31-40 % (5) | 41-50% (6) | >50 % (7) | N/A (8) |
| --- | --- | --- | --- | --- | --- | --- | --- | --- |
| Whole Carcass (1) |  |  |  |  |  |  |  |  |
| Parts (2) |  |  |  |  |  |  |  |  |
| Comminuted (4) |  |  |  |  |  |  |  |  |

Q190 What is your average whole carcass  <u><strong>Campylobacter</strong></u> prevalence per quarter (For calendar year starting January, 2021)

|  | Avg. Prevalence |  |  |  |  |  |  |  |
| --- | --- | --- | --- | --- | --- | --- | --- | --- |
|  | 0 % (1) | 1-10 % (2) | 11-20 % (3) | 21-30 % (4) | 31-40 % (5) | 41-50 % (6) | > 50 % (7) | N/A (8) |
| January-March (Winter) (5) |  |  |  |  |  |  |  |  |
| April-June (Spring) (6) |  |  |  |  |  |  |  |  |
| July-September (Summer) (7) |  |  |  |  |  |  |  |  |
| October-December (Fall) (8) |  |  |  |  |  |  |  |  |

Q191 What is your average parts  <u><strong>Campylobacter</strong></u> prevalence per quarter (For calendar year starting January, 2021)

|  | Avg. Prevalence |  |  |  |  |  |  |  |
| --- | --- | --- | --- | --- | --- | --- | --- | --- |
|  | 0 % (1) | 1-10 % (2) | 11-20 % (3) | 21-30 % (4) | 31-40 % (5) | 41-50 % (6) | > 50% (7) | N/A (8) |
| January-March (Winter) (5) |  |  |  |  |  |  |  |  |
| April-June (Spring) (6) |  |  |  |  |  |  |  |  |
| July-September (Summer) (7) |  |  |  |  |  |  |  |  |
| October-December (Fall) (8) |  |  |  |  |  |  |  |  |

Q192 What is your average comminuted <u><strong>Campylobacter</strong></u> prevalence per quarter (For calendar year starting January, 2021)

|  | Avg. Prevalence |  |  |  |  |  |  |  |
| --- | --- | --- | --- | --- | --- | --- | --- | --- |
|  | 0 % (1) | 1-10 % (2) | 11-20 % (3) | 21-30 % (4) | 31-40 % (5) | 41-50 % (6) | > 50% (7) | N/A (8) |
| January-March (Winter) (5) |  |  |  |  |  |  |  |  |
| April-June (Spring) (6) |  |  |  |  |  |  |  |  |
| July-September (Summer) (7) |  |  |  |  |  |  |  |  |
| October-December (Fall) (8) |  |  |  |  |  |  |  |  |

Q27 Are there any changes (i.e., new control strategy or intervention applied pre- or post-harvest) that have been implemented in your processing plant within the past calendar year to control <u><em><strong>Campylobacter</strong></em></u> and meet the performance standards for parts and proposed standards for comminuted product?

- Yes  (1) 

- No changes have been made regarding Campylobacter control in parts and comminuted product.  (2) 

- No, my establishment is already in compliance and no changes are needed.  (3) 

- No, my establishment is exempted from meeting the performance standards due to establishment type or size.  (4) 

Q28 What changes have you made in the past calendar year specifically to meet the performance standards for <u><em><strong>Campylobacter</strong></em></u> in parts and/or proposed standards for comminuted chicken or turkey?
<div> </div>

<div><strong><span style="font-size:13px;">Please check all that apply.</span></strong></div>

- Implement new on-farm interventions (such as vaccines, chemicals, prebiotics, and probiotics, and antibiotics) to reduce Campylobacter colonization in birds.   (1) 

- Implement or improve biosecurity and other management strategies on-farm to reduce Campylobacter colonization in birds   (2) 

- Implement on-farm microbiological testing to check Campylobacter prevalence or contamination levels.  (3) 

- Implement new post-harvest interventions in the processing facility to reduce Campylobacter contamination on the final product.   (4) 

- Implement in-plant microbiological testing to check Campylobacter contamination levels during broiler or turkey processing.  (5) 

- Other - Please specify your answer below.    (6) __________________________________________________

Q29 Does your processing establishment have validated interventions throughout second or further processing to <u>specifically</u> reduce <u><em><strong>Campylobacter</strong></em></u> contamination on product?

- Yes  (1) 

- No  (2) 

| Page Break |  |
| --- | --- |

Q73 Do you use Peroxyacetic Acid (PAA)?

- Yes  (1) 

- No  (2) 

| Page Break |  |
| --- | --- |

Q74 What type of equipment do you use to apply PAA? (select all that apply) 

- Spray Cabinets  (1) 

- Dip Tanks  (2) 

- Belts  (4) 

- Other  (3) __________________________________________________

| Page Break |  |
| --- | --- |

Q93 What is the PAA concentration in the ${q://QID75/ChoiceDescription/1}?

- 0-50 PPM  (1) 

- 51-100 PPM  (2) 

- 101-200 PPM  (4) 

- 201-300 PPM  (5) 

- 301-400 PPM  (6) 

- 401-500 PPM  (7) 

- 501-600 PPM  (8) 

- 601-700 PPM  (9) 

- 701-800 PPM  (10) 

- 801-900 PPM  (11) 

- 901-1000 PPM  (12) 

- 1001-1100 PPM  (13) 

- 1101-1200 PPM  (14) 

- 1201-1300 PPM  (15) 

- 1301-1400 PPM  (16) 

- 1401-1500 PPM  (17) 

- 1501-1600 PPM  (18) 

- 1601-1700 PPM  (19) 

- 1701-1800 PPM  (20) 

- 1801-1900 PPM  (21) 

- 1901-2000 PPM  (22) 

- > 2001 PPM  (23) 

Q94 What is your PAA contact time in the ${q://QID75/ChoiceDescription/1} ?

- 0 - 5 Seconds  (1) 

- 6-10 Seconds  (2) 

- 11-15 Seconds  (3) 

- 16-30 Seconds  (4) 

- 31-45 Seconds  (5) 

- 46-60 Seconds  (6) 

- 61-75 Seconds  (7) 

- 76-90 Seconds  (8) 

- > 90 Seconds  (9) 

Q95 What is your PAA solution pH in ${q://QID75/ChoiceDescription/1}

- 0-0.5  (1) 

- 0.6-1  (2) 

- 1.1-2  (3) 

- 2.1-3  (4) 

- 3.1-4  (5) 

- 4.1-5  (6) 

- 5.1-6  (7) 

- 6.1-7  (8) 

- 7.1-8  (9) 

- 8.1-9  (10) 

- 9.1-10  (11) 

- 10.1-11  (12) 

- 11.1-12  (13) 

- 12.1-13  (14) 

- 13.1-14  (15) 

- N/A  (16) 

| Page Break |  |
| --- | --- |

Q96 What is the PAA concentration in the ${q://QID75/ChoiceDescription/2} ?

- 0-50 PPM  (1) 

- 51-100 PPM  (2) 

- 101-200 PPM  (4) 

- 201-300 PPM  (5) 

- 301-400 PPM  (6) 

- 401-500 PPM  (7) 

- 501-600 PPM  (8) 

- 601-700 PPM  (9) 

- 701-800 PPM  (10) 

- 801-900 PPM  (11) 

- 901-1000 PPM  (12) 

- 1001-1100 PPM  (13) 

- 1101-1200 PPM  (14) 

- 1201-1300 PPM  (15) 

- 1301-1400 PPM  (16) 

- 1401-1500 PPM  (17) 

- 1501-1600 PPM  (18) 

- 1601-1700 PPM  (19) 

- 1701-1800 PPM  (20) 

- 1801-1900 PPM  (21) 

- 1901-2000 PPM  (22) 

- > 2001 PPM  (23) 

Q97 What is your PAA contact time in the ${q://QID75/ChoiceDescription/2}?

- 0 - 5 Seconds  (1) 

- 6-10 Seconds  (2) 

- 11-15 Seconds  (3) 

- 16-30 Seconds  (4) 

- 31-45 Seconds  (5) 

- 46-60 Seconds  (6) 

- 61-75 Seconds  (7) 

- 76-90 Seconds  (8) 

- > 90 Seconds  (9) 

Q98 What is your PAA solution pH in the ${q://QID75/ChoiceDescription/2} ?

- 0-0.5  (1) 

- 0.6-1  (2) 

- 1.1-2  (3) 

- 2.1-3  (4) 

- 3.1-4  (5) 

- 4.1-5  (6) 

- 5.1-6  (7) 

- 6.1-7  (8) 

- 7.1-8  (9) 

- 8.1-9  (10) 

- 9.1-10  (11) 

- 10.1-11  (12) 

- 11.1-12  (13) 

- 12.1-13  (14) 

- 13.1-14  (15) 

- N/A  (16) 

| Page Break |  |
| --- | --- |

Q99 What is the PAA concentration in the ${q://QID75/ChoiceDescription/4} ?

- 0-50 PPM  (1) 

- 51-100 PPM  (2) 

- 101-200 PPM  (4) 

- 201-300 PPM  (5) 

- 301-400 PPM  (6) 

- 401-500 PPM  (7) 

- 501-600 PPM  (8) 

- 601-700 PPM  (9) 

- 701-800 PPM  (10) 

- 801-900 PPM  (11) 

- 901-1000 PPM  (12) 

- 1001-1100 PPM  (13) 

- 1101-1200 PPM  (14) 

- 1201-1300 PPM  (15) 

- 1301-1400 PPM  (16) 

- 1401-1500 PPM  (17) 

- 1501-1600 PPM  (18) 

- 1601-1700 PPM  (19) 

- 1701-1800 PPM  (20) 

- 1801-1900 PPM  (21) 

- 1901-2000 PPM  (22) 

- > 2001 PPM  (23) 

Q100 What is your PAA contact time in the ${q://QID75/ChoiceDescription/4}?

- 0 - 5 Seconds  (1) 

- 6-10 Seconds  (2) 

- 11-15 Seconds  (3) 

- 16-30 Seconds  (4) 

- 31-45 Seconds  (5) 

- 46-60 Seconds  (6) 

- 61-75 Seconds  (7) 

- 76-90 Seconds  (8) 

- > 90 Seconds  (9) 

Q101 What is your PAA solution pH in the ${q://QID75/ChoiceDescription/4}?

- 0-0.5  (1) 

- 0.6-1  (2) 

- 1.1-2  (3) 

- 2.1-3  (4) 

- 3.1-4  (5) 

- 4.1-5  (6) 

- 5.1-6  (7) 

- 6.1-7  (8) 

- 7.1-8  (9) 

- 8.1-9  (10) 

- 9.1-10  (11) 

- 10.1-11  (12) 

- 11.1-12  (13) 

- 12.1-13  (14) 

- 13.1-14  (15) 

- N/A  (16) 

| Page Break |  |
| --- | --- |

Q102 What is the PAA concentration in the ${q://QID75/ChoiceTextEntryValue/3}?

- 0-50 PPM  (1) 

- 51-100 PPM  (2) 

- 101-200 PPM  (4) 

- 201-300 PPM  (5) 

- 301-400 PPM  (6) 

- 401-500 PPM  (7) 

- 501-600 PPM  (8) 

- 601-700 PPM  (9) 

- 701-800 PPM  (10) 

- 801-900 PPM  (11) 

- 901-1000 PPM  (12) 

- 1001-1100 PPM  (13) 

- 1101-1200 PPM  (14) 

- 1201-1300 PPM  (15) 

- 1301-1400 PPM  (16) 

- 1401-1500 PPM  (17) 

- 1501-1600 PPM  (18) 

- 1601-1700 PPM  (19) 

- 1701-1800 PPM  (20) 

- 1801-1900 PPM  (21) 

- 1901-2000 PPM  (22) 

- > 2001 PPM  (23) 

Q103 What is your PAA contact time in the ${q://QID75/ChoiceTextEntryValue/3}?

- 0 - 5 Seconds  (1) 

- 6-10 Seconds  (2) 

- 11-15 Seconds  (3) 

- 16-30 Seconds  (4) 

- 31-45 Seconds  (5) 

- 46-60 Seconds  (6) 

- 61-75 Seconds  (7) 

- 76-90 Seconds  (8) 

- > 90 Seconds  (9) 

Q104 What is your PAA solution pH in the ${q://QID75/ChoiceTextEntryValue/3}?

- 0-0.5  (1) 

- 0.6-1  (2) 

- 1.1-2  (3) 

- 2.1-3  (4) 

- 3.1-4  (5) 

- 4.1-5  (6) 

- 5.1-6  (7) 

- 6.1-7  (8) 

- 7.1-8  (9) 

- 8.1-9  (10) 

- 9.1-10  (11) 

- 10.1-11  (12) 

- 11.1-12  (13) 

- 12.1-13  (14) 

- 13.1-14  (15) 

- N/A  (16) 

| Page Break |  |
| --- | --- |

Q86 Do you use Acidified Sodium Chlorite (ASC)?

- Yes  (1) 

- No  (2) 

| Page Break |  |
| --- | --- |

Q87 What type of equipment do you use to apply ASC? (select all that apply)

- Spray Cabinets  (1) 

- Dip Tanks  (2) 

- Belts  (4) 

- Other  (3) __________________________________________________

| Page Break |  |
| --- | --- |

Q105 What is the ASC concentration in the ${q://QID88/ChoiceDescription/1}?

- 0-50 PPM  (1) 

- 51-100 PPM  (2) 

- 101-200 PPM  (4) 

- 201-300 PPM  (5) 

- 301-400 PPM  (6) 

- 401-500 PPM  (7) 

- 501-600 PPM  (8) 

- 601-700 PPM  (9) 

- 701-800 PPM  (10) 

- 801-900 PPM  (11) 

- 901-1000 PPM  (12) 

- 1001-1100 PPM  (13) 

- 1101-1200 PPM  (14) 

- >1201PPM  (15) 

Q106 What is your ASC contact time in the ${q://QID88/ChoiceDescription/1}?

- 0 - 5 Seconds  (1) 

- 6-10 Seconds  (2) 

- 11-15 Seconds  (3) 

- 16-30 Seconds  (4) 

- 31-45 Seconds  (5) 

- 46-60 Seconds  (6) 

- 61-75 Seconds  (7) 

- 76-90 Seconds  (8) 

- > 90 Seconds  (9) 

Q107 What is your ASC solution pH in the ${q://QID88/ChoiceDescription/1}?

- 0-0.5  (1) 

- 0.6-1  (2) 

- 1.1-2  (3) 

- 2.1-3  (4) 

- 3.1-4  (5) 

- 4.1-5  (6) 

- 5.1-6  (7) 

- 6.1-7  (8) 

- 7.1-8  (9) 

- 8.1-9  (10) 

- 9.1-10  (11) 

- 10.1-11  (12) 

- 11.1-12  (13) 

- 12.1-13  (14) 

- 13.1-14  (15) 

- N/A  (16) 

| Page Break |  |
| --- | --- |

Q108 What is the ASC concentration in the ${q://QID88/ChoiceDescription/2}?

- 0-50 PPM  (1) 

- 51-100 PPM  (2) 

- 101-200 PPM  (4) 

- 201-300 PPM  (5) 

- 301-400 PPM  (6) 

- 401-500 PPM  (7) 

- 501-600 PPM  (8) 

- 601-700 PPM  (9) 

- 701-800 PPM  (10) 

- 801-900 PPM  (11) 

- 901-1000 PPM  (12) 

- 1001-1100 PPM  (13) 

- 1101-1200 PPM  (14) 

- >1201PPM  (15) 

Q109 What is your ASC contact time in the ${q://QID88/ChoiceDescription/2}?

- 0 - 5 Seconds  (1) 

- 6-10 Seconds  (2) 

- 11-15 Seconds  (3) 

- 16-30 Seconds  (4) 

- 31-45 Seconds  (5) 

- 46-60 Seconds  (6) 

- 61-75 Seconds  (7) 

- 76-90 Seconds  (8) 

- > 90 Seconds  (9) 

Q110 What is your ASC solution pH in the ${q://QID88/ChoiceDescription/2}?

- 0-0.5  (1) 

- 0.6-1  (2) 

- 1.1-2  (3) 

- 2.1-3  (4) 

- 3.1-4  (5) 

- 4.1-5  (6) 

- 5.1-6  (7) 

- 6.1-7  (8) 

- 7.1-8  (9) 

- 8.1-9  (10) 

- 9.1-10  (11) 

- 10.1-11  (12) 

- 11.1-12  (13) 

- 12.1-13  (14) 

- 13.1-14  (15) 

- N/A  (16) 

| Page Break |  |
| --- | --- |

Q111 What is the ASC concentration in the ${q://QID88/ChoiceDescription/4}?

- 0-50 PPM  (1) 

- 51-100 PPM  (2) 

- 101-200 PPM  (4) 

- 201-300 PPM  (5) 

- 301-400 PPM  (6) 

- 401-500 PPM  (7) 

- 501-600 PPM  (8) 

- 601-700 PPM  (9) 

- 701-800 PPM  (10) 

- 801-900 PPM  (11) 

- 901-1000 PPM  (12) 

- 1001-1100 PPM  (13) 

- 1101-1200 PPM  (14) 

- >1201PPM  (15) 

Q112 What is your ASC contact time in the ${q://QID88/ChoiceDescription/4}?

- 0 - 5 Seconds  (1) 

- 6-10 Seconds  (2) 

- 11-15 Seconds  (3) 

- 16-30 Seconds  (4) 

- 31-45 Seconds  (5) 

- 46-60 Seconds  (6) 

- 61-75 Seconds  (7) 

- 76-90 Seconds  (8) 

- > 90 Seconds  (9) 

Q113 What is your ASC solution pH in the ${q://QID88/ChoiceDescription/4}?

- 0-0.5  (1) 

- 0.6-1  (2) 

- 1.1-2  (3) 

- 2.1-3  (4) 

- 3.1-4  (5) 

- 4.1-5  (6) 

- 5.1-6  (7) 

- 6.1-7  (8) 

- 7.1-8  (9) 

- 8.1-9  (10) 

- 9.1-10  (11) 

- 10.1-11  (12) 

- 11.1-12  (13) 

- 12.1-13  (14) 

- 13.1-14  (15) 

- N/A  (16) 

| Page Break |  |
| --- | --- |

Q114 What is the ASC concentration in the ${q://QID88/ChoiceTextEntryValue/3}?

- 0-50 PPM  (1) 

- 51-100 PPM  (2) 

- 101-200 PPM  (4) 

- 201-300 PPM  (5) 

- 301-400 PPM  (6) 

- 401-500 PPM  (7) 

- 501-600 PPM  (8) 

- 601-700 PPM  (9) 

- 701-800 PPM  (10) 

- 801-900 PPM  (11) 

- 901-1000 PPM  (12) 

- 1001-1100 PPM  (13) 

- 1101-1200 PPM  (14) 

- >1201PPM  (15) 

Q115 What is your ASC contact time in the ${q://QID88/ChoiceTextEntryValue/3}?

- 0 - 5 Seconds  (1) 

- 6-10 Seconds  (2) 

- 11-15 Seconds  (3) 

- 16-30 Seconds  (4) 

- 31-45 Seconds  (5) 

- 46-60 Seconds  (6) 

- 61-75 Seconds  (7) 

- 76-90 Seconds  (8) 

- > 90 Seconds  (9) 

Q116 What is your ASC solution pH in the ${q://QID88/ChoiceTextEntryValue/3}?

- 0-0.5  (1) 

- 0.6-1  (2) 

- 1.1-2  (3) 

- 2.1-3  (4) 

- 3.1-4  (5) 

- 4.1-5  (6) 

- 5.1-6  (7) 

- 6.1-7  (8) 

- 7.1-8  (9) 

- 8.1-9  (10) 

- 9.1-10  (11) 

- 10.1-11  (12) 

- 11.1-12  (13) 

- 12.1-13  (14) 

- 13.1-14  (15) 

- N/A  (16) 

| Page Break |  |
| --- | --- |

Q117 Do you use Chlorine (Measured as Free Available Chlorine)?

- Yes  (1) 

- No  (2) 

| Page Break |  |
| --- | --- |

Q118 What type of equipment do you use to apply Chlorine? (select all that apply)

- Spray Cabinets  (1) 

- Dip Tanks  (2) 

- Belts  (4) 

- Other  (3) __________________________________________________

| Page Break |  |
| --- | --- |

Q120 What is the Chlorine (free available chlorine) concentration in the ${q://QID119/ChoiceDescription/1}?

- 0 PPM  (1) 

- 5 PPM  (2) 

- 10 PPM  (4) 

- 20 PPM  (5) 

- 30 PPM  (6) 

- 40 PPM  (7) 

- 50 PPM  (8) 

- 60 PPM  (9) 

- 70 PPM  (10) 

- 80 PPM  (11) 

- 90 PPM  (12) 

- 100 PPM  (13) 

- 110 PPM  (14) 

- 120 PPM  (15) 

- 130 PPM  (24) 

- 140 PPM  (25) 

- 150 PPM  (26) 

- 160 PPM  (27) 

- 170 PPM  (28) 

- 180 PPM  (29) 

- 190 PPM  (30) 

- 200 PPM  (31) 

- >201 PPM  (32) 

Q121 What is your Chlorine contact time in the ${q://QID119/ChoiceDescription/1}?

- 0 - 5 Seconds  (1) 

- 6-10 Seconds  (2) 

- 11-15 Seconds  (3) 

- 16-30 Seconds  (4) 

- 31-45 Seconds  (5) 

- 46-60 Seconds  (6) 

- 61-75 Seconds  (7) 

- 76-90 Seconds  (8) 

- > 90 Seconds  (9) 

Q122 What is your Chlorine solution pH in the ${q://QID119/ChoiceDescription/1}?

- 0-0.5  (1) 

- 0.6-1  (2) 

- 1.1-2  (3) 

- 2.1-3  (4) 

- 3.1-4  (5) 

- 4.1-5  (6) 

- 5.1-6  (7) 

- 6.1-7  (8) 

- 7.1-8  (9) 

- 8.1-9  (10) 

- 9.1-10  (11) 

- 10.1-11  (12) 

- 11.1-12  (13) 

- 12.1-13  (14) 

- 13.1-14  (15) 

- N/A  (16) 

| Page Break |  |
| --- | --- |

Q123 What is the Chlorine (free available chlorine) concentration in the ${q://QID119/ChoiceDescription/2}?

- 0 PPM  (1) 

- 5 PPM  (2) 

- 10 PPM  (4) 

- 20 PPM  (5) 

- 30 PPM  (6) 

- 40 PPM  (7) 

- 50 PPM  (8) 

- 60 PPM  (9) 

- 70 PPM  (10) 

- 80 PPM  (11) 

- 90 PPM  (12) 

- 100 PPM  (13) 

- 110 PPM  (14) 

- 120 PPM  (15) 

- 130 PPM  (24) 

- 140 PPM  (25) 

- 150 PPM  (26) 

- 160 PPM  (27) 

- 170 PPM  (28) 

- 180 PPM  (29) 

- 190 PPM  (30) 

- 200 PPM  (31) 

- >201 PPM  (32) 

Q124 What is your Chlorine contact time in the ${q://QID119/ChoiceDescription/2}?

- 0 - 5 Seconds  (1) 

- 6-10 Seconds  (2) 

- 11-15 Seconds  (3) 

- 16-30 Seconds  (4) 

- 31-45 Seconds  (5) 

- 46-60 Seconds  (6) 

- 61-75 Seconds  (7) 

- 76-90 Seconds  (8) 

- > 90 Seconds  (9) 

Q125 What is your Chlorine solution pH in the ${q://QID119/ChoiceDescription/2}?

- 0-0.5  (1) 

- 0.6-1  (2) 

- 1.1-2  (3) 

- 2.1-3  (4) 

- 3.1-4  (5) 

- 4.1-5  (6) 

- 5.1-6  (7) 

- 6.1-7  (8) 

- 7.1-8  (9) 

- 8.1-9  (10) 

- 9.1-10  (11) 

- 10.1-11  (12) 

- 11.1-12  (13) 

- 12.1-13  (14) 

- 13.1-14  (15) 

- N/A  (16) 

| Page Break |  |
| --- | --- |

Q126 What is the Chlorine (free available chlorine) concentration in the ${q://QID119/ChoiceDescription/4}?

- 0 PPM  (1) 

- 5 PPM  (2) 

- 10 PPM  (4) 

- 20 PPM  (5) 

- 30 PPM  (6) 

- 40 PPM  (7) 

- 50 PPM  (8) 

- 60 PPM  (9) 

- 70 PPM  (10) 

- 80 PPM  (11) 

- 90 PPM  (12) 

- 100 PPM  (13) 

- 110 PPM  (14) 

- 120 PPM  (15) 

- 130 PPM  (24) 

- 140 PPM  (25) 

- 150 PPM  (26) 

- 160 PPM  (27) 

- 170 PPM  (28) 

- 180 PPM  (29) 

- 190 PPM  (30) 

- 200 PPM  (31) 

- >201 PPM  (32) 

Q127 What is your Chlorine contact time in the ${q://QID119/ChoiceDescription/4}?

- 0 - 5 Seconds  (1) 

- 6-10 Seconds  (2) 

- 11-15 Seconds  (3) 

- 16-30 Seconds  (4) 

- 31-45 Seconds  (5) 

- 46-60 Seconds  (6) 

- 61-75 Seconds  (7) 

- 76-90 Seconds  (8) 

- > 90 Seconds  (9) 

Q128 What is your Chlorine solution pH in the ${q://QID119/ChoiceDescription/4}?

- 0-0.5  (1) 

- 0.6-1  (2) 

- 1.1-2  (3) 

- 2.1-3  (4) 

- 3.1-4  (5) 

- 4.1-5  (6) 

- 5.1-6  (7) 

- 6.1-7  (8) 

- 7.1-8  (9) 

- 8.1-9  (10) 

- 9.1-10  (11) 

- 10.1-11  (12) 

- 11.1-12  (13) 

- 12.1-13  (14) 

- 13.1-14  (15) 

- N/A  (16) 

| Page Break |  |
| --- | --- |

Q129 What is the Chlorine (free available chlorine) concentration in the ${q://QID119/ChoiceTextEntryValue/3}?

- 0 PPM  (1) 

- 5 PPM  (2) 

- 10 PPM  (4) 

- 20 PPM  (5) 

- 30 PPM  (6) 

- 40 PPM  (7) 

- 50 PPM  (8) 

- 60 PPM  (9) 

- 70 PPM  (10) 

- 80 PPM  (11) 

- 90 PPM  (12) 

- 100 PPM  (13) 

- 110 PPM  (14) 

- 120 PPM  (15) 

- 130 PPM  (24) 

- 140 PPM  (25) 

- 150 PPM  (26) 

- 160 PPM  (27) 

- 170 PPM  (28) 

- 180 PPM  (29) 

- 190 PPM  (30) 

- 200 PPM  (31) 

- >201 PPM  (32) 

Q130 What is your Chlorine contact time in the ${q://QID119/ChoiceTextEntryValue/3}?

- 0 - 5 Seconds  (1) 

- 6-10 Seconds  (2) 

- 11-15 Seconds  (3) 

- 16-30 Seconds  (4) 

- 31-45 Seconds  (5) 

- 46-60 Seconds  (6) 

- 61-75 Seconds  (7) 

- 76-90 Seconds  (8) 

- > 90 Seconds  (9) 

Q131 What is your Chlorine solution pH in the ${q://QID119/ChoiceTextEntryValue/3}?

- 0-0.5  (1) 

- 0.6-1  (2) 

- 1.1-2  (3) 

- 2.1-3  (4) 

- 3.1-4  (5) 

- 4.1-5  (6) 

- 5.1-6  (7) 

- 6.1-7  (8) 

- 7.1-8  (9) 

- 8.1-9  (10) 

- 9.1-10  (11) 

- 10.1-11  (12) 

- 11.1-12  (13) 

- 12.1-13  (14) 

- 13.1-14  (15) 

- N/A  (16) 

| Page Break |  |
| --- | --- |

Q132 Do you use Cetylpyridinium chloride (CPC)?

- Yes  (1) 

- No  (2) 

| Page Break |  |
| --- | --- |

Q133 What type of equipment do you use to apply CPC? (select all that apply)

- Spray Cabinets  (1) 

- Dip Tanks  (2) 

- Belts  (4) 

- Other  (3) __________________________________________________

| Page Break |  |
| --- | --- |

Q134 What is the CPC concentration in the ${q://QID134/ChoiceDescription/1}?

- 0 g/lb  (2) 

- 0.1 g/lb  (4) 

- 0.2 g/lb  (5) 

- 0.3 g/lb  (6) 

- >0.31 g/lb  (7) 

Q135 What is your CPC contact time in the ${q://QID134/ChoiceDescription/1}?

- 0 - 5 Seconds  (1) 

- 6-10 Seconds  (2) 

- 11-15 Seconds  (3) 

- 16-30 Seconds  (4) 

- 31-45 Seconds  (5) 

- 46-60 Seconds  (6) 

- 61-75 Seconds  (7) 

- 76-90 Seconds  (8) 

- > 90 Seconds  (9) 

Q136 What is your CPC solution pH in the ${q://QID134/ChoiceDescription/1}?

- 0-0.5  (1) 

- 0.6-1  (2) 

- 1.1-2  (3) 

- 2.1-3  (4) 

- 3.1-4  (5) 

- 4.1-5  (6) 

- 5.1-6  (7) 

- 6.1-7  (8) 

- 7.1-8  (9) 

- 8.1-9  (10) 

- 9.1-10  (11) 

- 10.1-11  (12) 

- 11.1-12  (13) 

- 12.1-13  (14) 

- 13.1-14  (15) 

- N/A  (16) 

| Page Break |  |
| --- | --- |

Q137 What is the CPC concentration in the ${q://QID134/ChoiceDescription/2}?

- 0 g/lb  (2) 

- 0.1 g/lb  (4) 

- 0.2 g/lb  (5) 

- 0.3 g/lb  (6) 

- >0.31 g/lb  (7) 

Q138 What is your CPC contact time in the ${q://QID134/ChoiceDescription/2}?

- 0 - 5 Seconds  (1) 

- 6-10 Seconds  (2) 

- 11-15 Seconds  (3) 

- 16-30 Seconds  (4) 

- 31-45 Seconds  (5) 

- 46-60 Seconds  (6) 

- 61-75 Seconds  (7) 

- 76-90 Seconds  (8) 

- > 90 Seconds  (9) 

Q139 What is your CPC solution pH in the ${q://QID134/ChoiceDescription/2}?

- 0-0.5  (1) 

- 0.6-1  (2) 

- 1.1-2  (3) 

- 2.1-3  (4) 

- 3.1-4  (5) 

- 4.1-5  (6) 

- 5.1-6  (7) 

- 6.1-7  (8) 

- 7.1-8  (9) 

- 8.1-9  (10) 

- 9.1-10  (11) 

- 10.1-11  (12) 

- 11.1-12  (13) 

- 12.1-13  (14) 

- 13.1-14  (15) 

- N/A  (16) 

| Page Break |  |
| --- | --- |

Q142 What is the CPC concentration in the ${q://QID134/ChoiceDescription/4}?

- 0 g/lb  (2) 

- 0.1 g/lb  (4) 

- 0.2 g/lb  (5) 

- 0.3 g/lb  (6) 

- >0.31 g/lb  (7) 

Q143 What is your CPC contact time in the ${q://QID134/ChoiceDescription/4}?

- 0 - 5 Seconds  (1) 

- 6-10 Seconds  (2) 

- 11-15 Seconds  (3) 

- 16-30 Seconds  (4) 

- 31-45 Seconds  (5) 

- 46-60 Seconds  (6) 

- 61-75 Seconds  (7) 

- 76-90 Seconds  (8) 

- > 90 Seconds  (9) 

Q144 What is your CPC solution pH in the ${q://QID134/ChoiceDescription/4}?

- 0-0.5  (1) 

- 0.6-1  (2) 

- 1.1-2  (3) 

- 2.1-3  (4) 

- 3.1-4  (5) 

- 4.1-5  (6) 

- 5.1-6  (7) 

- 6.1-7  (8) 

- 7.1-8  (9) 

- 8.1-9  (10) 

- 9.1-10  (11) 

- 10.1-11  (12) 

- 11.1-12  (13) 

- 12.1-13  (14) 

- 13.1-14  (15) 

- N/A  (16) 

| Page Break |  |
| --- | --- |

Q145 What is the CPC concentration in the ${q://QID134/ChoiceTextEntryValue/3}?

- 0 g/lb  (2) 

- 0.1 g/lb  (4) 

- 0.2 g/lb  (5) 

- 0.3 g/lb  (6) 

- >0.31 g/lb  (7) 

Q146 What is your CPC contact time in the ${q://QID134/ChoiceTextEntryValue/3}?

- 0 - 5 Seconds  (1) 

- 6-10 Seconds  (2) 

- 11-15 Seconds  (3) 

- 16-30 Seconds  (4) 

- 31-45 Seconds  (5) 

- 46-60 Seconds  (6) 

- 61-75 Seconds  (7) 

- 76-90 Seconds  (8) 

- > 90 Seconds  (9) 

Q147 What is your CPC solution pH in the ${q://QID134/ChoiceTextEntryValue/3}?

- 0-0.5  (1) 

- 0.6-1  (2) 

- 1.1-2  (3) 

- 2.1-3  (4) 

- 3.1-4  (5) 

- 4.1-5  (6) 

- 5.1-6  (7) 

- 6.1-7  (8) 

- 7.1-8  (9) 

- 8.1-9  (10) 

- 9.1-10  (11) 

- 10.1-11  (12) 

- 11.1-12  (13) 

- 12.1-13  (14) 

- 13.1-14  (15) 

- N/A  (16) 

| Page Break |  |
| --- | --- |

Q148 Do you use Chlorine Dioxide?

- Yes  (1) 

- No  (2) 

| Page Break |  |
| --- | --- |

Q149 What type of equipment do you use to apply Chlorine Dioxide? (select all that apply)

- Spray Cabinets  (1) 

- Dip Tanks  (2) 

- Belts  (4) 

- Other  (3) __________________________________________________

| Page Break |  |
| --- | --- |

Q150 What is the Chlorine Dioxide concentration in the ${q://QID150/ChoiceDescription/1}?

- 0 PPM  (2) 

- 1 PPM  (4) 

- 2 PPM  (5) 

- 3 PPM  (6) 

- > 3 PPM  (7) 

Q151 What is your Chlorine Dioxide contact time in the ${q://QID150/ChoiceDescription/1}?

- 0 - 5 Seconds  (1) 

- 6-10 Seconds  (2) 

- 11-15 Seconds  (3) 

- 16-30 Seconds  (4) 

- 31-45 Seconds  (5) 

- 46-60 Seconds  (6) 

- 61-75 Seconds  (7) 

- 76-90 Seconds  (8) 

- > 90 Seconds  (9) 

Q152 What is your Chlorine Dioxide solution pH in the ${q://QID150/ChoiceDescription/1}?

- 0-0.5  (1) 

- 0.6-1  (2) 

- 1.1-2  (3) 

- 2.1-3  (4) 

- 3.1-4  (5) 

- 4.1-5  (6) 

- 5.1-6  (7) 

- 6.1-7  (8) 

- 7.1-8  (9) 

- 8.1-9  (10) 

- 9.1-10  (11) 

- 10.1-11  (12) 

- 11.1-12  (13) 

- 12.1-13  (14) 

- 13.1-14  (15) 

- N/A  (16) 

| Page Break |  |
| --- | --- |

Q153 What is the Chlorine Dioxide concentration in the ${q://QID150/ChoiceDescription/2}?

- 0 PPM  (2) 

- 1 PPM  (4) 

- 2 PPM  (5) 

- 3 PPM  (6) 

- > 3 PPM  (7) 

Q154 What is your Chlorine Dioxide contact time in the ${q://QID150/ChoiceDescription/2}?

- 0 - 5 Seconds  (1) 

- 6-10 Seconds  (2) 

- 11-15 Seconds  (3) 

- 16-30 Seconds  (4) 

- 31-45 Seconds  (5) 

- 46-60 Seconds  (6) 

- 61-75 Seconds  (7) 

- 76-90 Seconds  (8) 

- > 90 Seconds  (9) 

Q155 What is your Chlorine Dioxide solution pH in the ${q://QID150/ChoiceDescription/2}?

- 0-0.5  (1) 

- 0.6-1  (2) 

- 1.1-2  (3) 

- 2.1-3  (4) 

- 3.1-4  (5) 

- 4.1-5  (6) 

- 5.1-6  (7) 

- 6.1-7  (8) 

- 7.1-8  (9) 

- 8.1-9  (10) 

- 9.1-10  (11) 

- 10.1-11  (12) 

- 11.1-12  (13) 

- 12.1-13  (14) 

- 13.1-14  (15) 

- N/A  (16) 

| Page Break |  |
| --- | --- |

Q156 What is the Chlorine Dioxide concentration in the ${q://QID150/ChoiceDescription/4}?

- 0 PPM  (2) 

- 1 PPM  (4) 

- 2 PPM  (5) 

- 3 PPM  (6) 

- > 3 PPM  (7) 

Q157 What is your Chlorine Dioxide contact time in the ${q://QID150/ChoiceDescription/4}?

- 0 - 5 Seconds  (1) 

- 6-10 Seconds  (2) 

- 11-15 Seconds  (3) 

- 16-30 Seconds  (4) 

- 31-45 Seconds  (5) 

- 46-60 Seconds  (6) 

- 61-75 Seconds  (7) 

- 76-90 Seconds  (8) 

- > 90 Seconds  (9) 

Q158 What is your Chlorine Dioxide solution pH in the ${q://QID150/ChoiceDescription/4}?

- 0-0.5  (1) 

- 0.6-1  (2) 

- 1.1-2  (3) 

- 2.1-3  (4) 

- 3.1-4  (5) 

- 4.1-5  (6) 

- 5.1-6  (7) 

- 6.1-7  (8) 

- 7.1-8  (9) 

- 8.1-9  (10) 

- 9.1-10  (11) 

- 10.1-11  (12) 

- 11.1-12  (13) 

- 12.1-13  (14) 

- 13.1-14  (15) 

- N/A  (16) 

| Page Break |  |
| --- | --- |

Q159 What is the Chlorine Dioxide concentration in the ${q://QID150/ChoiceTextEntryValue/3}?

- 0 PPM  (2) 

- 1 PPM  (4) 

- 2 PPM  (5) 

- 3 PPM  (6) 

- > 3 PPM  (7) 

Q160 What is your Chlorine Dioxide contact time in the ${q://QID150/ChoiceTextEntryValue/3}?

- 0 - 5 Seconds  (1) 

- 6-10 Seconds  (2) 

- 11-15 Seconds  (3) 

- 16-30 Seconds  (4) 

- 31-45 Seconds  (5) 

- 46-60 Seconds  (6) 

- 61-75 Seconds  (7) 

- 76-90 Seconds  (8) 

- > 90 Seconds  (9) 

Q161 What is your Chlorine Dioxide solution pH in the ${q://QID150/ChoiceTextEntryValue/3}?

- 0-0.5  (1) 

- 0.6-1  (2) 

- 1.1-2  (3) 

- 2.1-3  (4) 

- 3.1-4  (5) 

- 4.1-5  (6) 

- 5.1-6  (7) 

- 6.1-7  (8) 

- 7.1-8  (9) 

- 8.1-9  (10) 

- 9.1-10  (11) 

- 10.1-11  (12) 

- 11.1-12  (13) 

- 12.1-13  (14) 

- 13.1-14  (15) 

- N/A  (16) 

| Page Break |  |
| --- | --- |

Q162 Do you use Trisodium phosphate (TSP)?

- Yes  (1) 

- No  (2) 

| Page Break |  |
| --- | --- |

Q163 What type of equipment do you use to apply TSP? (select all that apply)

- Spray Cabinets  (1) 

- Dip Tanks  (2) 

- Belts  (4) 

- Other  (3) __________________________________________________

| Page Break |  |
| --- | --- |

Q164 What is the TSP concentration in the ${q://QID164/ChoiceDescription/1}?

- 0.0%  (2) 

- 0.1-0.5%  (4) 

- 0.6-1.0%  (5) 

- 1.1-2%  (6) 

- 2.1-3%  (7) 

- 3.1-4%  (33) 

- 4.1-5%  (34) 

- 5.1-6%  (35) 

- 6.1-7%  (36) 

- 7.1-8%  (37) 

- 8.1-9%  (38) 

- 9.1-10%  (39) 

- 10.1-11%  (40) 

- 11.1-12%  (41) 

- >12%  (42) 

Q165 What is your TSP contact time in the ${q://QID164/ChoiceDescription/1}?

- 0 - 5 Seconds  (1) 

- 6-10 Seconds  (2) 

- 11-15 Seconds  (3) 

- 16-30 Seconds  (4) 

- 31-45 Seconds  (5) 

- 46-60 Seconds  (6) 

- 61-75 Seconds  (7) 

- 76-90 Seconds  (8) 

- > 90 Seconds  (9) 

Q166 What is your TSP solution pH in the ${q://QID164/ChoiceDescription/1}?

- 0-0.5  (1) 

- 0.6-1  (2) 

- 1.1-2  (3) 

- 2.1-3  (4) 

- 3.1-4  (5) 

- 4.1-5  (6) 

- 5.1-6  (7) 

- 6.1-7  (8) 

- 7.1-8  (9) 

- 8.1-9  (10) 

- 9.1-10  (11) 

- 10.1-11  (12) 

- 11.1-12  (13) 

- 12.1-13  (14) 

- 13.1-14  (15) 

- N/A  (16) 

| Page Break |  |
| --- | --- |

Q167 What is the TSP concentration in the ${q://QID164/ChoiceDescription/2}?

- 0.0%  (2) 

- 0.1-0.5%  (4) 

- 0.6-1.0%  (5) 

- 1.1-2%  (6) 

- 2.1-3%  (7) 

- 3.1-4%  (33) 

- 4.1-5%  (34) 

- 5.1-6%  (35) 

- 6.1-7%  (36) 

- 7.1-8%  (37) 

- 8.1-9%  (38) 

- 9.1-10%  (39) 

- 10.1-11%  (40) 

- 11.1-12%  (41) 

- >12%  (42) 

Q168 What is your TSP contact time in the ${q://QID164/ChoiceDescription/2}?

- 0 - 5 Seconds  (1) 

- 6-10 Seconds  (2) 

- 11-15 Seconds  (3) 

- 16-30 Seconds  (4) 

- 31-45 Seconds  (5) 

- 46-60 Seconds  (6) 

- 61-75 Seconds  (7) 

- 76-90 Seconds  (8) 

- > 90 Seconds  (9) 

Q169 What is your TSP solution pH in the ${q://QID164/ChoiceDescription/2}?

- 0-0.5  (1) 

- 0.6-1  (2) 

- 1.1-2  (3) 

- 2.1-3  (4) 

- 3.1-4  (5) 

- 4.1-5  (6) 

- 5.1-6  (7) 

- 6.1-7  (8) 

- 7.1-8  (9) 

- 8.1-9  (10) 

- 9.1-10  (11) 

- 10.1-11  (12) 

- 11.1-12  (13) 

- 12.1-13  (14) 

- 13.1-14  (15) 

- N/A  (16) 

| Page Break |  |
| --- | --- |

Q170 What is the TSP concentration in the ${q://QID164/ChoiceDescription/4}?

- 0.0%  (2) 

- 0.1-0.5%  (4) 

- 0.6-1.0%  (5) 

- 1.1-2%  (6) 

- 2.1-3%  (7) 

- 3.1-4%  (33) 

- 4.1-5%  (34) 

- 5.1-6%  (35) 

- 6.1-7%  (36) 

- 7.1-8%  (37) 

- 8.1-9%  (38) 

- 9.1-10%  (39) 

- 10.1-11%  (40) 

- 11.1-12%  (41) 

- >12%  (42) 

Q171 What is your TSP contact time in the ${q://QID164/ChoiceDescription/4}?

- 0 - 5 Seconds  (1) 

- 6-10 Seconds  (2) 

- 11-15 Seconds  (3) 

- 16-30 Seconds  (4) 

- 31-45 Seconds  (5) 

- 46-60 Seconds  (6) 

- 61-75 Seconds  (7) 

- 76-90 Seconds  (8) 

- > 90 Seconds  (9) 

Q172 What is your TSP solution pH in the ${q://QID164/ChoiceDescription/4}?

- 0-0.5  (1) 

- 0.6-1  (2) 

- 1.1-2  (3) 

- 2.1-3  (4) 

- 3.1-4  (5) 

- 4.1-5  (6) 

- 5.1-6  (7) 

- 6.1-7  (8) 

- 7.1-8  (9) 

- 8.1-9  (10) 

- 9.1-10  (11) 

- 10.1-11  (12) 

- 11.1-12  (13) 

- 12.1-13  (14) 

- 13.1-14  (15) 

- N/A  (16) 

| Page Break |  |
| --- | --- |

Q173 What is the TSP concentration in the ${q://QID164/ChoiceTextEntryValue/3}?

- 0.0%  (2) 

- 0.1-0.5%  (4) 

- 0.6-1.0%  (5) 

- 1.1-2%  (6) 

- 2.1-3%  (7) 

- 3.1-4%  (33) 

- 4.1-5%  (34) 

- 5.1-6%  (35) 

- 6.1-7%  (36) 

- 7.1-8%  (37) 

- 8.1-9%  (38) 

- 9.1-10%  (39) 

- 10.1-11%  (40) 

- 11.1-12%  (41) 

- >12%  (42) 

Q174 What is your TSP contact time in the ${q://QID164/ChoiceTextEntryValue/3}?

- 0 - 5 Seconds  (1) 

- 6-10 Seconds  (2) 

- 11-15 Seconds  (3) 

- 16-30 Seconds  (4) 

- 31-45 Seconds  (5) 

- 46-60 Seconds  (6) 

- 61-75 Seconds  (7) 

- 76-90 Seconds  (8) 

- > 90 Seconds  (9) 

Q175 What is your TSP solution pH in the ${q://QID164/ChoiceTextEntryValue/3}?

- 0-0.5  (1) 

- 0.6-1  (2) 

- 1.1-2  (3) 

- 2.1-3  (4) 

- 3.1-4  (5) 

- 4.1-5  (6) 

- 5.1-6  (7) 

- 6.1-7  (8) 

- 7.1-8  (9) 

- 8.1-9  (10) 

- 9.1-10  (11) 

- 10.1-11  (12) 

- 11.1-12  (13) 

- 12.1-13  (14) 

- 13.1-14  (15) 

- N/A  (16) 

| Page Break |  |
| --- | --- |

Q176 Do you use Ultraviolet light on parts and comminuted poultry?

- Yes  (1) 

- No  (2) 

Q177 Do you use Ozone on parts and comminuted poultry?

- Yes  (23) 

- No  (24) 

| Page Break |  |
| --- | --- |

Q32 Does your processing establishment test incoming poultry from external suppliers for <u><em><strong>Campylobacter</strong></em></u>?

- Yes  (1) 

- No  (2) 

Q33 Does your processing establishment require external suppliers of poultry to test source materials for <strong><em><u>Campylobacter</u></em></strong>?

- Yes  (1) 

- No  (2) 

Q34 In which of the following categories does the external supplier of poultry typically fall into with respect to the FSIS performance standards for <u><em><strong>Campylobacter</strong></em></u>?

- Category 1    (1) 

- Category 2    (2) 

- Category 3    (3) 

- Don't know    (4) 

End of Block: Block 2

Start of Block: Block 3

Q35 <div><strong><span style="font-size:19px;">Pre-Harvest Campylobacter Control</span></strong></div>

<div> </div>
The following questions collects background information about <strong><u>on-farm</u></strong> procedures targeting <u><em><strong>Campylobacter</strong></em></u> contamination on carcasses.

| Page Break |  |
| --- | --- |

Q36 Which of the following on-farm interventions or management practices have been implemented in the past calendar year specifically to reduce <u><em><strong>Campylobacter</strong></em></u> colonization in broiler chickens and/or turkeys?<br>
<br>
<span style="font-size:13px;"><strong>Please check all that apply.</strong></span>

- Improvements in biosecurity  (1) 

- Change in drinking water program   (2) 

- Change litter management program   (3) 

- Administration of antibiotics  (4) 

- Administration of feed supplements such as prebiotics and/or probiotics  (5) 

- Other:  (7) __________________________________________________

- None  (6) 

Q37 Is the <u><em><strong>Campylobacter </strong></em></u>contamination status of incoming flocks known prior to slaughter?

- Yes, microbiological test results are available for every incoming flock    (1) 

- Yes, microbiological test results are available for a subset of incoming flocks   (2) 

- No   (3) 

Q38 Do you measure <u><em><strong>Campylobacter</strong></em></u> for incoming flocks?

- Yes  (1) 

- No  (2) 

Q40 What is your average incoming <u><strong>Campylobacter</strong></u> positive flocks per quarter (For calendar year starting January, 2021)

|  | Avg. Percent Positive Flocks |  |  |  |  |  |  |  |  |  |  |  |
| --- | --- | --- | --- | --- | --- | --- | --- | --- | --- | --- | --- | --- |
|  | 0 % (1) | 1-10 % (2) | 11-20 % (3) | 21-30 % (4) | 31-40 % (5) | 41-50 % (6) | 51-60 % (7) | 61-70 % (8) | 71-80 % (9) | 81-90 % (10) | 91-100 % (11) | N/A (12) |
| January-March (Winter) (5) |  |  |  |  |  |  |  |  |  |  |  |  |
| April-June (Spring) (6) |  |  |  |  |  |  |  |  |  |  |  |  |
| July-September (Summer) (7) |  |  |  |  |  |  |  |  |  |  |  |  |
| October-December (Fall) (8) |  |  |  |  |  |  |  |  |  |  |  |  |

Q41 Do you use this data for logistic slaughter (i.e., are Campylobacter-free flocks processed before Campylobacter-positive flocks)?

- Yes  (1) 

- No  (2) 

Q42 Do you use this data to determine product disposition (i.e., send meat from Campylobacter-positive flocks to a cooked product line)?

- Yes  (1) 

- No  (2) 

Q43 Are transportation modules cleaned between flocks?

- Yes    (1) 

- No   (2) 

- I do not know because contract live haul crews are responsible for cleaning modules   (3) 

End of Block: Block 3

Start of Block: Block 5

Q64 <span style="font-size:19px;"><strong>First-Processing Campylobacter Control</strong></span><br />
<br />
The following questions collects background information about <strong><u>first processing </u></strong>procedures targeting <u><em><strong>Campylobacter</strong></em></u> contamination on carcasses.

| Page Break |  |
| --- | --- |

Q19 Where do you take samples for <u><em><strong>Campylobacter</strong></em></u> in First Processing?
<div><strong><span style="font-size:13px;">Please check all that apply</span></strong></div>

- Live Haul  (1) 

- Hot Re-hang  (2) 

- Evisceration  (3) 

- Pre-Chill  (4) 

- Post-Chill  (5) 

- Other (please specify):  (6) __________________________________________________

- None of the above  (8) 

Q44 Which of the following scalding methods is currently being used?

- Immersion scalding  (1) 

- Steam spraying scalding   (2) 

Q71 How long have you used this scalding method?

- Less than 1 year  (1) 

- 1 year or more  (2) 

Q46 Which of the following procedures are currently being used during <strong>immersion</strong> scalding and how long have they been used?

|  | Less than 1 year	 (1) | 1 year or more (2) | Not used (3) |
| --- | --- | --- | --- |
| Pre-scald brush system (1) |  |  |  |
| Counter flow of process water  (2) |  |  |  |
| Multiple stage tanks  (3) |  |  |  |
| Administration of interventions to scald water (e.g. PAA, salt, adjust pH)  (4) |  |  |  |
| Post-scald wash  (5) |  |  |  |
| Administration of interventions to post-scald wash (e.g. PAA, salt, adjust pH)  (6) |  |  |  |

Q47 Which of the following procedures are currently being used during <strong>steam</strong> scalding and how long have they been used?

|  | Less than 1 year (1) | 1 year or more (2) | Not used (3) |
| --- | --- | --- | --- |
| Pre-scald brush system (1) |  |  |  |
| Post-scald wash (2) |  |  |  |
| Administration of interventions to post-scald wash (e.g. PAA, salt, adjust pH) (3) |  |  |  |

Q48 Is product meeting FSIS performance standards for <u><em><strong>Campylobacter </strong></em></u>after scalding procedures?

- Yes    (1) 

- No    (2) 

- N/A    (3) 

Q49 Which of the following procedures are currently being used during <strong>picking</strong> and how long have they been used?

|  | Less than 1 year	 (1) | 1 year or more	 (2) | Not used (3) |
| --- | --- | --- | --- |
| Continuous rinsing of carcasses during picking (1) |  |  |  |
| Continuous rinsing of equipment during picking (2) |  |  |  |
| Post-pick application of chemicals or antimicrobial agents (in wash, dip, or other manner)  (3) |  |  |  |

Q50 Is product meeting FSIS performance standards for <u><em><strong>Campylobacter</strong></em></u> after picking procedures?

- Yes    (1) 

- No    (2) 

- N/A    (3) 

Q189 Is product meeting FSIS performance standards for <u><em><strong>Campylobacter </strong></em></u>at hot-rehang?

- Yes  (1) 

- No  (2) 

- N/A  (3) 

Q51 Which of the following procedures are currently being used during <b>evisceration</b> and how long have they been used?

|  | Less than 1 year	 (1) | 1 year or more	 (2) | Not used (3) |
| --- | --- | --- | --- |
| Automated re-hang (1) |  |  |  |
| Daily replacement of opener blades (2) |  |  |  |
| Whole carcass rinse during evisceration (3) |  |  |  |
| Post-evisceration wash (4) |  |  |  |
| Application of chemicals or antimicrobial agents in post-evisceration wash (5) |  |  |  |

Q52 Is product meeting FSIS performance standards for <u><em><strong>Campylobacter</strong></em></u> after evisceration procedures?

- Yes    (1) 

- No    (2) 

- N/A    (3) 

Q54 Which <strong>chilling </strong>method are you currently using?

- Air chilling   (1) 

- Immersion chilling   (2) 

Q72 How long has this chilling method been used?

- Less than 1 year  (1) 

- 1 year or longer  (2) 

Q56 Which of the following procedures are currently being used during <strong>immersion chilling </strong>and how long have they been used?

|  | Less than 1 year	 (1) | 1 year or more (2) | Not used (3) |
| --- | --- | --- | --- |
| Use of pre-chill tanks  (1) |  |  |  |
| Antimicrobial treatment in pre-chill tanks (2) |  |  |  |
| Use of counter-current flow during chilling (3) |  |  |  |
| Addition of chlorine in chill water  (4) |  |  |  |
| Addition of other antimicrobial agents in chill water (5) |  |  |  |
| Use of Oxidation Reduction Potential (ORP) meter (6) |  |  |  |
| Use of post-chill tanks (7) |  |  |  |
| Antimicrobial treatment in post-chill tanks (8) |  |  |  |

Q57 Which of the following procedures are currently being used during <strong>air chilling </strong>and how long have they been used?

|  | Less than 1 year	 (1) | 1 year or more (2) | Not used (3) |
| --- | --- | --- | --- |
| Use of a pre-chill stage  (1) |  |  |  |
| Antimicrobial treatment in a pre-chill stage  (2) |  |  |  |
| Addition of chlorine in chill air  (3) |  |  |  |
| Regular maintenance of chains and shackles  (4) |  |  |  |

Q59 Is product meeting FSIS performance standards for <u><em><strong>Campylobacter</strong></em></u> after chilling procedures?

- Yes   (1) 

- No   (2) 

- N/A   (3) 

Q178 Which antimicrobial do you use in pre-chill tanks?

- Peroxyacetic acid (PAA)  (1) 

- Acidified sodium chlorite  (2) 

- Cetylpyridinium chloride (CPC)  (3) 

- Chlorine (Free Available Chlorine)  (4) 

- Chlorine dioxide  (5) 

- Trisodium phospate  (6) 

- Other  (7) __________________________________________________

- N/A  (8) 

Q179 Which antimicrobial do you use in immersion chillers?

- Peroxyacetic acid (PAA)  (1) 

- Acidified sodium chlorite  (2) 

- Cetylpyridinium chloride (CPC)  (3) 

- Chlorine (Free Available Chlorine)  (4) 

- Chlorine dioxide  (5) 

- Trisodium phospate  (6) 

- Other  (7) __________________________________________________

- N/A  (8) 

Q182 Which antimicrobial do you use in post-chill tanks?

- Peroxyacetic acid (PAA)  (1) 

- Acidified sodium chlorite  (2) 

- Cetylpyridinium chloride (CPC)  (3) 

- Chlorine (Free Available Chlorine)  (4) 

- Chlorine dioxide  (5) 

- Trisodium phospate  (6) 

- Other  (7) __________________________________________________

- N/A  (8) 

End of Block: Block 5

Start of Block: Block 4

Q62 Additional Comments:<div><br></div><div>This section provides space to enter any additional information not covered in the previous questions. </div>

| Page Break |  |
| --- | --- |

Q60 Enter any additional information about Campylobacter control that can be shared and it is not covered in this survey.

________________________________________________________________

Q61 Enter any other comment in the space below.

________________________________________________________________

End of Block: Block 4
